# A Trust-Boundary Reference Architecture for Secure Cyber-Physical Agriculture: Design and Software-Based Proof-of-Concept for Smart Irrigation

**DOI:** 10.3390/s26206545

**Published:** 2026-10-15

**Authors:** Alvertos Ioannis Mourikis, Iosif Polenakis, Christos Sarantidis, Romanos Kalamatianos, Ioannis Karydis

**Affiliations:** Department of Informatics, Ionian University, 49100 Corfu, Greece; amourikis@ionio.gr (A.I.M.); ipolenakis@ionio.gr (I.P.); rkalam@ionio.gr (R.K.); karydis@ionio.gr (I.K.)

**Keywords:** smart irrigation, digital farming, operational technology, cyber-physical systems, trust boundaries, threat modelling, provenance, edge control, sensor integrity, secure lifecycle

## Abstract

Smart irrigation is an operational technology (OT) problem as much as an Internet of Things (IoT) one: software decisions act directly on valves, pumps, pressure and water delivery, so a spoofed reading or a replayed command has physical consequences. This paper presents a technology-neutral reference architecture that makes those consequences controllable. It is organised into seven functional layers and three cross-cutting planes, six trust-boundary classes that fix which properties must be re-established at each crossing, and an auditable crown-asset inventory with an explicit trust-level rubric. Two governing execution principles enforce this structure: local safety authority, which keeps final actuation permission at the field gateway, remote terminal unit (RTU) or programmable logic controller (PLC), and *feedback-aware execution*, which treats a command as complete only when independent physical feedback confirms it. Building on process-aware and safety-supervision ideas from industrial control, the architecture makes physical confirmation a mandatory completion condition at the local enforcement point, which catches a fully authorised command that is nonetheless physically unsafe, a failure mode that authorisation checks alone cannot detect. These properties are examined through a deterministic, openly available software proof-of-concept that verifies the design against its own acceptance criteria; it is not a field validation. The software executes six attack and failure scenarios plus one nominal-operation control against an intentionally unsecured negative control and a secure controller, under identical, controlled conditions: the negative control is compromised in all 6 attack scenarios and the secure controller in 0/6, with 0/1 false positives on legitimate operation. Analytically, the design costs 21 bytes and 50–60% additional Long Range Wide Area Network (LoRaWAN) time-on-air per record, and 78% of single-step perturbations of the trust-scoring inputs leave asset classification unchanged: the actuator class (A2) is completely stable, and the gateway/RTU/PLC class (A3) is stable under 6 of 7 admissible perturbations (86%), changing only when its credential/update authority reaches the trust-anchor threshold. A sensor-level mapping from sampling rate, measurement drift and physical fault modes to the edge-verification controls they dictate, provisions for secure time, counter recovery and the choice between fail-closed and fail-open safe states, an illustrative hypothetical worked deployment example, and a transfer of the model to greenhouse, fertigation, livestock-watering and cold-chain settings show how the architecture could be applied in practice.

## 1. Introduction

Secure digital agriculture should not be viewed only as a cloud application that collects measurements from field sensors. In irrigation control, sensors, valves, pumps, local controllers, dashboards, and cloud services form a cyber-physical system (CPS) whose digital decisions affect the physical environment and the production process [1,2,3]. Consequently, the security objective is broader than confidentiality or network protection. It also includes reliability, availability, operational safety, auditability, resilience, and the ability to maintain a bounded safe state under degraded conditions [1,4,5].

Nassi et al. [6] showed that a botnet of commercial irrigation controllers could open valves in concert and empty an urban water tower overnight, an attack in which no data is stolen and the entire damage is hydraulic. Sontowski et al. [7] demonstrated on a smart-farm testbed that inexpensive wireless deauthentication and spoofing silence or falsify the soil-moisture stream a scheduler depends on, so the controller acts confidently on measurements that no longer describe the field. Pasca et al. [8] report the same weakness as a design property rather than an accident: agricultural nodes routinely ship without device authentication or replay protection, so a recorded packet stays valid indefinitely; a systematic review confirms these findings are representative [9]. The consequences are physical: a season of suppressed irrigation, a pump destroyed by dry running, a field waterlogged by a command replayed at 3 a.m., a fertigation dose delivered twice, and none is prevented by encrypting the telemetry, because in every case the message is well formed and the channel intact. What fails is the decision to act on it.

Such failures are physical and economic rather than informational: a spoofed soil-moisture value suppresses irrigation when water is needed, a replayed command waters without authorisation, a stale weather feed misleads a scheduler, and a compromised dashboard token turns a legitimate interface into an unsafe override. Trustworthiness, therefore, depends less on encryption than on *where* measurements are produced, commands authorised, actuation physically enforced, and evidence preserved. A reference architecture must consequently integrate three viewpoints: the layered structure of smart agriculture, from field devices to governance [10,11,12,13]; the separation of data, control, and management planes, whose flows carry different assurance requirements [1,14,15] and the identification of trust boundaries, where assumptions change about identity, authority, administrative control, freshness, exposure, or physical protection [1,4,14].

Two design principles follow and govern the model. *Local safety authority*: cloud analytics, dashboards, and decision-support systems may generate schedules, recommendations, or override requests, but final permission to actuate a valve, pump, relay, or variable-frequency drive (VFD) stays with the local gateway, remote terminal unit (RTU), or programmable logic controller (PLC)-like controller, which verifies that a command is authorised, fresh, within policy, compatible with interlocks, and safe under current conditions before execution [1,2,12]. *Feedback-aware execution*: a command is successful not because software sent or accepted it, but only once independent physical feedback (flow, pressure, valve state, or soil-moisture response) confirms it. A trust boundary here need not be a firewall or network hop; it is any location where data, instructions, permissions, settings, or audit records enter a new trust relationship, and every crossing must re-establish authentication, authorisation, freshness, integrity, and auditability in proportion to the risk borne by the receiving function.

The literature reviewed in Section 1.1 reveals three gaps, substantiated in Table 1. First, smart-agriculture architectures list layers and components but do not define where trust assumptions change and which controls must be re-enforced there. Second, security studies describe attacks or controls without connecting them to irrigation-specific crown assets (pumps, valves, gateways, command queues, signing keys, operator overrides). Third, conceptual architectures lack scenario-based acceptance criteria that make them testable. This paper closes those gaps through an architecture-level synthesis translating operational technology (OT) security guidance, Internet of Things (IoT) baselines, provenance reasoning, and irrigation-control requirements into one reusable design and evaluation framework. It contributes a technology-neutral reference architecture organised by functional layers and by data, control, and management planes, in which six trust-boundary classes each carry an explicit obligation and an irrigation-centric crown-asset inventory assigns every critical class a trust level, safety impact, misuse modes, and minimum controls. It converts those requirements into testable acceptance criteria through a scenario-based threat model executed by a deterministic, openly available proof-of-concept, and characterises quantitatively both what the design costs, through per-message and airtime overhead analysis, and how robust its judgements are, through perturbation analysis of the trust rubric. Finally, it grounds the model in physical sensing, mapping sampling rate, drift, and hardware fault modes onto the controls they dictate, and illustrates the procedure on a hypothetical worked deployment before generalising to adjacent settings.

Concretely, this paper makes the following contributions:

- a technology-neutral reference architecture organised by seven functional layers and three cross-cutting planes;
- six trust-boundary classes (B1–B6), each with an explicit, per-crossing re-establishment obligation, derived traceably from nine requirements distilled from OT, IoT and irrigation-control sources (Section 2.2);
- an irrigation-centric crown-asset inventory (A1–A6) with an auditable trust-level rubric, its decision procedure, and a quantified sensitivity analysis of that procedure;
- two design principles, local safety authority and feedback-aware execution, formalised as a machine-checkable telemetry-provenance model;
- a scenario-based set of falsifiable acceptance criteria, exercised by a deterministic, openly available proof-of-concept, together with an analytical cost and sensitivity characterisation and a worked, illustrative deployment example.

Each contribution is one of three kinds: a synthesis of existing standards and requirements into a taxonomy (the architecture, boundaries and asset inventory); a deterministic, code-checked specification (the proof-of-concept); or an analytical calculation, from the published Long Range (LoRa) time-on-air model and the stated record structure (the overhead figures) or from the trust rubric itself (the sensitivity figures). Section 2.1 states once how results of these kinds should be read.

The paper is organised as follows. Section 1.1 reviews the four strands of related work and positions the architecture against them in tabular form. Section 2 presents the architecture and formal model: derivation methodology, the mapping from standards to layers and boundaries, the layered architecture and its planes, the boundary classes, the crown-asset inventory, the formalisation of the two design principles with the telemetry-provenance model, and the sensor-level integration. Section 3 gives the threat model, based on the STRIDE categories (spoofing, tampering, repudiation, information disclosure, denial of service, elevation of privilege), and discusses lifecycle governance, interoperability, and operational cost. Section 4 reports the results; Section 5 interprets them, compares the framework against existing reference architectures, and states the limitations, while in Section 6 we conclude our work.

### 1.1. Related Work

This section positions the proposed architecture within four strands of literature, i.e., cyber-physical smart agriculture, edge and operational-technology control, threat modelling, and provenance and lifecycle security, and closes with a positioning subsection that substantiates, in tabular form, the three gaps stated above.

#### 1.1.1. Cyber-Physical Systems in Smart Agriculture

The transition toward Agriculture 4.0 has replaced gravity-driven irrigation with automated, data-driven configurations in which soil-moisture sensors and automated valves close a loop around computational intelligence. Systematic reviews of cyber-physical systems for smart farming [16] and of smart farming technologies [17] document the shift, as do accounts of CPS-driven productivity [18] and of Industry 4.0 principles in wider cyber-physical settings [19]. Within irrigation, Qian et al. [20] survey the cyber-physical perspective, Ali et al. [21] assess water-use efficiency, and Perea et al. [22] predict irrigation events using optimal decision trees.

IoT deployment underpins the real-time data collection that precise intervention requires, in smart greenhouses [23], in industrial cyber-physical systems [24], in cyber-agricultural systems for crop breeding [25], and in greenhouse energy-efficiency control [26]. The tight coupling of computational logic to physical components is itself the vulnerability, which is why robust security assessment is repeatedly identified as necessary [27]. Related control-oriented work, greenhouse simulation [28], disturbance-observer sliding-mode control [29], and hardware-in-the-loop microclimate stabilisation [30], establishes the closed-loop dynamics that the security controls proposed here must not disrupt.

#### 1.1.2. Edge Control and Operational Technology

Edge computing reduces bandwidth demand and cloud dependency: Baseca et al. [31] combine Long Range Wide Area Network (LoRaWAN) connectivity with long short-term memory networks for predictive deficit irrigation, Liu and Cai [32] secure traceability through an edge-cloud-blockchain framework, and Dumitrache et al. [33] formalise design across cloud and edge paradigms; surveys cover cyber-physical-social systems [34] and CPS design techniques [35]. Processing telemetry locally enables real-time commands without continuous connectivity. The cost is that localised OT commonly runs on legacy protocols lacking native encryption or authentication [36], motivating lightweight secure elements for exposed rural nodes and tamper-resistant [37] and privacy-preserving [38] authentication schemes.

#### 1.1.3. Threat Modelling and Trust-Boundary Analysis

Structured threat modelling becomes imperative where farming networks bridge the physical and digital domains. Vakhnovskyi [39] performs targeted threat modelling and attack-surface analysis for IoT-enabled controlled-environment agriculture. Complementing adversarial analysis, model-based safety analysis using fault-tree analysis predicts component-failure propagation in IoT irrigation systems and lets fail-safe mechanisms be designed into the conceptual topology, as Abdulhamid et al. [40] demonstrate for a smart irrigation network.

A further body of industrial-control work checks commands and process behaviour against physical expectations rather than against credentials alone, and is directly related to feedback-aware execution. Safety instrumented systems implement independent protective layers that act on measured process variables irrespective of the commanding software [41]; command-supervision and safety-verification approaches check controller logic against safety properties of the plant before it takes effect [42]; process-aware intrusion detection monitors the semantics of controller variables and commands [43]; invariant-based detection checks relationships that must hold among sensor and actuator states, as demonstrated on a water-treatment testbed [44] and physics-based anomaly detection compares observed measurements with a model of the controlled process [45,46,47]. This work establishes that physical consistency is a recognised security signal. It typically operates as a detection or protection layer alongside the command path; however, rather than making physical confirmation a mandatory completion condition for each individual command at the local enforcement point, it is tied to the trust boundary through which the command arrived and to the evidence recorded for it. That integration, rather than the underlying idea, is what the present architecture contributes.

#### 1.1.4. Provenance, Authentication, and Secure Lifecycle

Telemetry integrity and device identity are foundational. Against man-in-the-middle attacks and data poisoning, blockchain and decentralised identifiers establish tamper-evident provenance: Elmatsani et al. [48] address governance in agricultural smart contracts, Manoj et al. [49] combine blockchain with trusted federated learning, and Aliyu [50] proposes a smart-farm framework against data poisoning; a wider agri-food literature establishes the same requirement in adjacent settings [51,52,53,54,55,56]. Secure lifecycle management additionally depends on safe over-the-air firmware updates, for which Noprianto et al. [57] propose a mechanism based on continuous integration and continuous delivery (CI/CD).

#### 1.1.5. Positioning in the Literature

The closest works perform structured threat or safety analysis of agricultural IoT [39,40] or adapt industrial standards to agriculture [4]. Vakhnovskyi [39] maps the attack surface of controlled-environment agriculture but does not connect the threats to a layered architecture or asset-level trust classification. Abdulhamid et al. [40] use fault-tree analysis for component failure in a smart-irrigation network, complementary to but narrower than a trust-boundary treatment. Shaaban et al. [4] map International Electrotechnical Commission (IEC) 62443 security levels onto agricultural devices, but device-centrically rather than boundary-centrically. Zero-Trust Architecture work in industrial settings [58,59] shares the premise that no crossing is trusted by default; the present paper narrows that principle to irrigation flows and ties it to a concrete local-enforcement mechanism rather than a general access-control posture.

**Figure 1:**
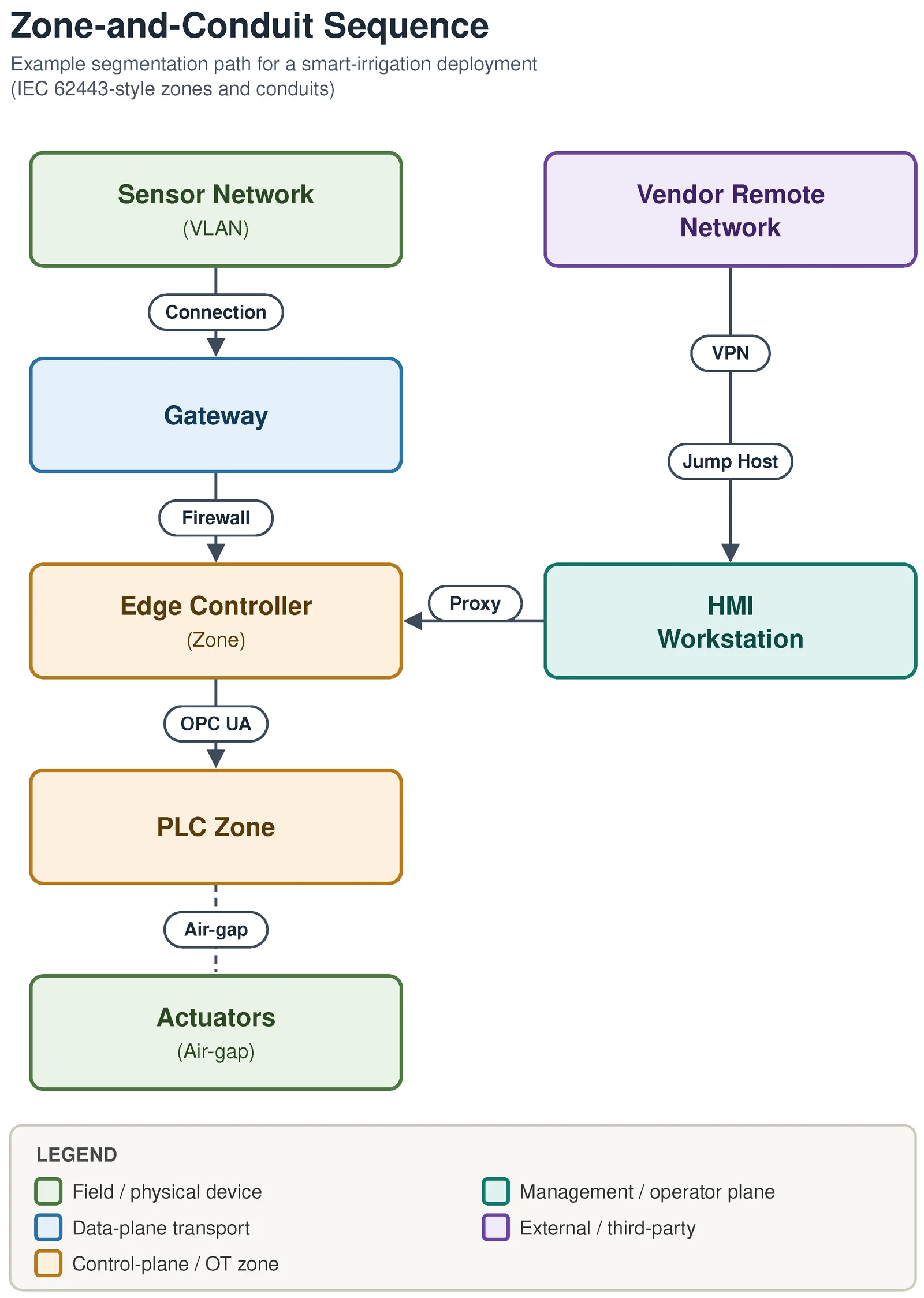
Example zone-and-conduit sequence for a smart-irrigation deployment. Each transition can require different controls and logging assumptions.

The relation to IEC 62443 zones and conduits deserves explicit statement, since both partition a system into regions with defined communication paths. A zones-and-conduits model [4], illustrated in Figure 1 answers *where* a boundary sits and *what security level* a zone must satisfy, but is agnostic to which property must be re-checked when a payload crosses it. The B1–B6 model of Table 2 instead attaches a payload-aware obligation to each class. A dashboard-to-gateway command (B3) and a weather-feed-to-scheduler input (B6) may traverse the same zone/conduit pair yet need materially different controls, dual approval and session binding versus corroboration based on a time-to-live (TTL), a distinction a conduit mapping cannot make once the two share a conduit. The schemes are, therefore, complementary: segmentation layers beneath the boundary classes, which determine the obligations for whatever traffic a conduit carries.

Table 3 compares the architecture with the closest existing approaches along the dimensions that matter for irrigation safety: an explicit layered architecture, trust boundaries as first-class objects with per-crossing obligations, asset classification by authority over the physical process, actuation conditional on local enforcement and independent feedback, machine-readable provenance, and testable acceptance criteria.

Two observations follow. First, no existing approach combines a layered architecture with per-crossing trust obligations and asset-level authority classification; approaches strong on one dimension are silent on the others. Second, and more important for irrigation, none of the approaches in Table 3 addresses feedback-aware execution explicitly, and the industrial-control row is rated *partially or implicitly addressed* because it uses physical consistency as a detection or protection signal rather than as a completion condition: they treat a command as complete once authorised, whereas this architecture treats it as complete only once the hydraulic state confirms it. Related ideas exist in adjacent industrial-control work, in safety instrumented systems, process-aware intrusion detection, control invariants and physics-based anomaly detection (Section 1.1.3), but as detection or protection layers alongside the command path rather than as a per-command completion condition integrated with boundary-specific authorisation and evidence. We, therefore, claim novelty for that integration, not for the idea of physical verification itself. Section 4.2 shows the distinction is not cosmetic, since one of six scenarios is defeated by feedback verification alone, after every authorisation check has passed.

Table 1 summarises, per strand, what the literature establishes, what it leaves open for secure irrigation, and how this paper responds, making the derivation from literature to model explicit and traceable.

Two of the responses recorded there, carry the weight of the later evaluation and are, therefore, named here before they are used: the STRIDE asset×boundary matrix of Table 4, which places every threat at the crossing where it must be answered, and the scenario-based acceptance criteria of Table 5, which state what evidence would falsify the design. The first is developed in Section 3.1 and the second in Section 4.1.

## 2. Reference Architecture and Formal Model

This section presents the architecture and the model governing its behaviour, in the order in which the model is built: the derivation methodology, the traceable mapping from standards and requirements to layers and boundary classes, the layered architecture and its three planes, the six boundary classes and their enforcement patterns, the crown-asset inventory specifying what is protected, the two design principles and the machine-checkable provenance model that enforce those guarantees, and the sensor-level integration grounding the model in physical sensing.

### 2.1. Methodology

The proposed architecture was developed through a qualitative, architecture-driven methodology. The objective was not to optimise a specific farm deployment or evaluate a single protocol. Instead, the objective was to derive a reusable reference model that can be mapped to heterogeneous irrigation deployments while preserving safety-relevant security properties. The methodology contains six steps.

The paper is a design-science contribution [60]: it builds an artefact, the reference architecture of Section 2.3, Section 2.4, Section 2.5, Section 2.6 and Section 2.7, which in design-science terms is a model and a method, and evaluates it by the methods appropriate to a pre-deployment stage of that paradigm, namely structured argument, formal specification, a deterministic executable model, and analytical calculation. Accordingly, *verification* is used throughout in its requirements-engineering sense: checking, under controlled and deterministic injection, that the specified controls satisfy the architecture’s own falsifiable acceptance criteria (Section 4.1). It is not used in the sense of *validation*, i.e., demonstrating the performance of an installed system under real sensing, weather and hydraulic conditions, which is out of scope. Every quantitative figure in Section 4, whether a scenario outcome, an overhead figure or a sensitivity figure, is, therefore, a property of the authors’ executable specification or an analytical calculation from stated parameters, not an empirical measurement on a field system. This statement applies to all later sections and is not repeated there; its consequences are stated once, in Section 5.4.

**Figure 2:**
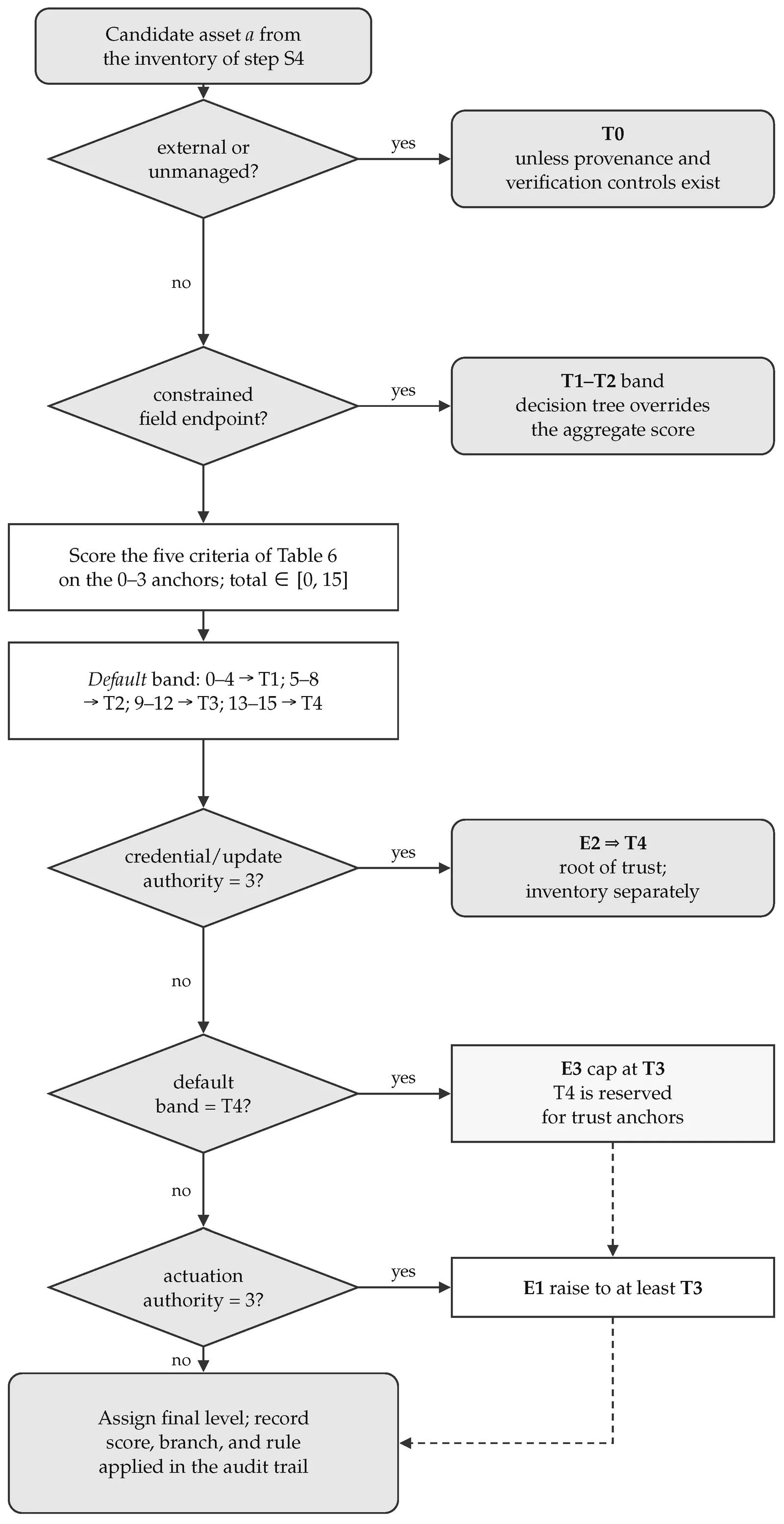
Decision flow for assigning a crown-asset trust level. The entry tests for external and constrained-endpoint assets take precedence over the aggregate score; the score then yields a default band, which the adjudication rules E2 (escalate to T4), E3 (cap at T3) and E1 (raise to at least T3) correct in that order. The branch taken is recorded as part of the classification audit trail.

S1. *Concept and standards synthesis.* Principles were synthesised from OT security, IoT device baselines, secure software development, smart-agriculture architectures, irrigation-control literature, and provenance-oriented reasoning [1,2,10,14,61], identifying the core requirements of trustworthy irrigation: identity, authorisation, integrity, freshness, provenance, auditability, local enforcement, resilience, and secure lifecycle management. Procedurally, this was a structured targeted review, not a systematic review in the sense of the Preferred Reporting Items for Systematic Reviews and Meta-Analyses (PRISMA), and we say so explicitly. Normative sources, such as National Institute of Standards and Technology (NIST) Special Publication (SP) 800-82r3, SP 800-160v1r1 and Interagency/Internal Report (IR) 8425, IEC 62443 as applied to agriculture, International Organisation for Standardisation (ISO)/IEC 27400 [62] and 27402 [63], and the European Telecommunications Standards Institute (ETSI) consumer-IoT baseline [64,65], were included for the control families they define; peer-reviewed sources were retrieved from Scopus, IEEE Xplore and the MDPI corpus, primarily for 2016–2026 using combinations of *smart irrigation*, *agricultural IoT*, *cyber-physical*, *operational technology*, *trust boundary*, *threat model* and *provenance* [66]. The search was performed manually in these databases, and no contemporaneous log of records returned, screened or excluded was kept; selection and exclusion were, therefore, author-driven, based on relevance to the layers, boundaries and assets of the architecture, and the review is not reproducible as a systematic search. Foundational works outside this window (on soil physics, design science and industrial-control security), normative documents, and a small number of preprints were added by backward citation chasing where they are the original source of a concept used here; preprints are identified as such in the reference list. To keep the route from sources to requirements inspectable, Table 6 lists the principal sources behind each of R1–R9. Candidates were merged where two sources expressed the same obligation and retained only where each could be tied to an observable irrigation failure mode, yielding the nine requirements R1–R9. No expert workshops or interviews were held; all judgements are the authors’, a limitation restated in Section 5.4.
S2. *Architectural decomposition.* The ecosystem was decomposed into functional layers separating field devices, actuators, local controllers, connectivity, platform services, applications, and governance, then examined through three cross-cutting planes. Two functions belong to different layers when they differ in authority over the physical process or in resource and exposure profile, and to different planes when one asset handles them under different assurance requirements, since a single gateway forwards telemetry, enforces commands, and receives firmware. A layer was retained only if at least one requirement is discharged at it.
S3. *Trust-boundary identification.* Boundary classes were defined by asking where assumptions change about identity, administrative control, exposure, physical access, freshness, or accountability, producing the six classes defined in Table 2. The procedure ran in three passes: enumerate every directed inter-layer flow on each plane; annotate each candidate crossing with the subset of six trust attributes whose assumptions change and merge candidates with identical signatures, since a crossing is defined by what must be re-established rather than by topology. B1–B6 are the resulting equivalence classes, which is why the taxonomy has six members, and why two flows sharing a conduit but differing in signature remain distinct.
S4. *Crown-asset inventory.* Assets were classified by authority over the physical process, trust level, resource constraints, and misuse modes. Candidates were enumerated by listing every component appearing as an endpoint of at least one crossing from step S3, then grouped where they share both a trust level and a minimum control set, collapsing all soil-moisture, flow and pressure transducers into class A1. Each class was scored on five criteria, mapped to a default band, and adjudicated by three precedence rules; criteria and rules are in Table 7 and the procedure in Figure 2, so any level in Table 8 can be re-derived, with stability quantified in Table 9.
S5. *Threat and control mapping.* Attacks and failures were mapped to assets, boundaries, impacts, and controls under a STRIDE-informed logic tailored to irrigation safety rather than software-only threats. The six asset classes were crossed exhaustively with the six boundary classes and all 36 cells populated with dominant STRIDE categories and an impact rating against fixed anchors, giving the matrix of Table 4; exhaustive population is what makes an absence meaningful, since a cell with no plausible threat is a claim rather than an omission.
S6. *Scenario-based verification.* The architecture was translated into the testable scenarios of Table 5, each defining an injection, the expected response, the evidence to record, and an acceptance criterion. It provides a structured method for evaluating whether an implementation preserves the architecture’s assumptions. Scenarios were derived from the step-S5 matrix by a minimality-and-coverage rule and each was implemented as executable code, so the criterion is evaluated by machine rather than asserted. The rule selects the smallest injection set that exercises every boundary class, involves every asset class, isolates one enforcement mechanism per scenario, and covers the dominant STRIDE categories.

**Figure 3:**
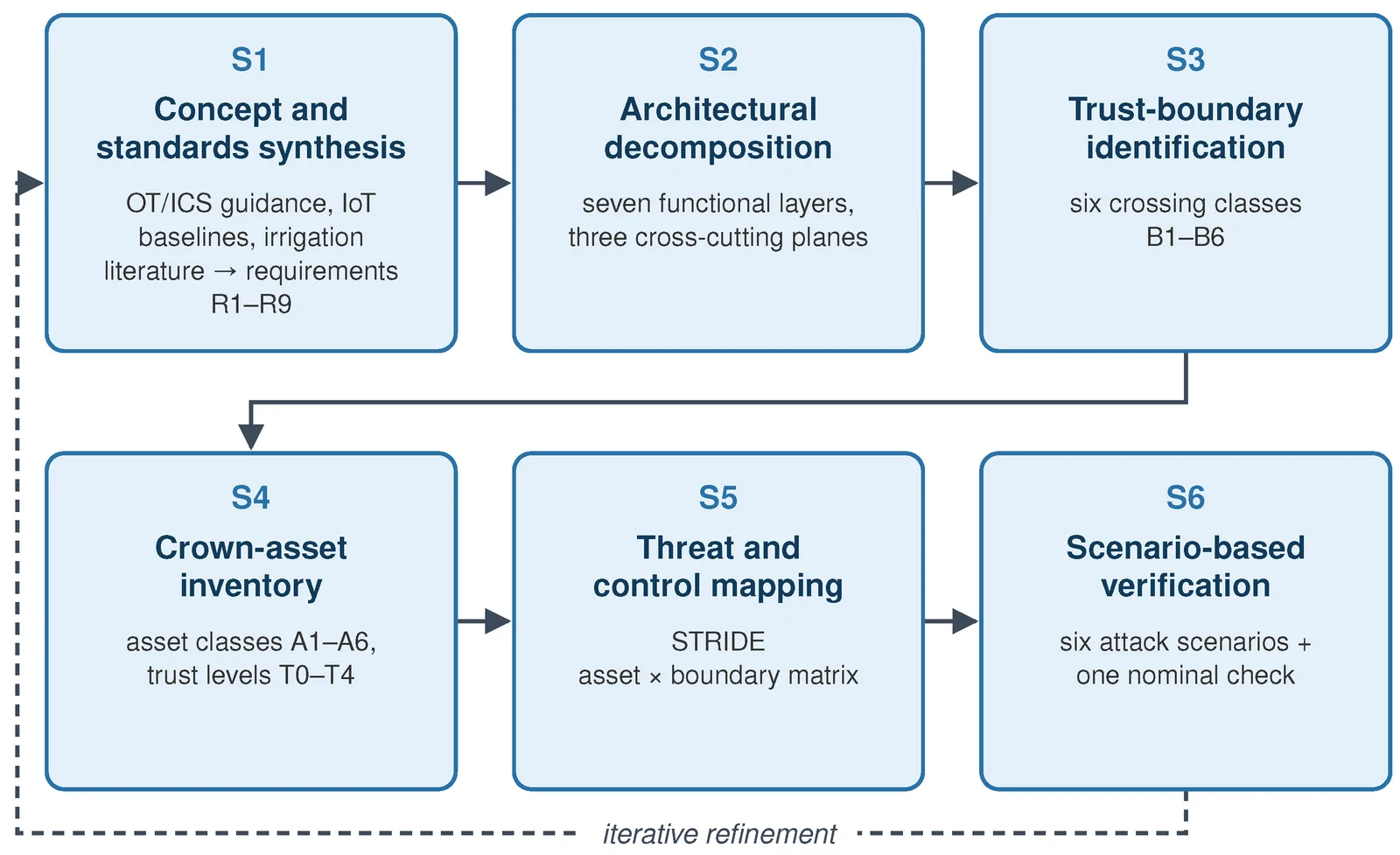
Methodology used to derive the reference architecture. Steps S1–S6 are executed in sequence, with an iterative refinement loop in which threat mapping and scenario construction feed unmet obligations back into the architectural decomposition and boundary definitions.

Figure 3 summarises the process. The steps are sequential on a first pass but iterative in practice: threat mapping (S5) and scenario construction (S6) repeatedly exposed missing obligations at particular crossings, which fed back into steps S2 and S3. The nominal-operation scenario of Section 4.2, for instance, was added only after the first verification pass showed that six attack scenarios establish sensitivity but not specificity.

### 2.2. From Standards and Requirements to Layers and Boundaries

A reference architecture is only as defensible as the reasoning behind it; thus, this section states the nine requirements distilled in step S1 and shows the layer and boundary class at which each is discharged: no layer or boundary class exists because it is conventional.

The requirements are as follows: R1, unique, verifiable device and workload identity; R2, message integrity and origin authentication; R3, freshness and replay resistance; R4, authorisation proportionate to the physical authority of the receiving function; R5, local enforcement of safety-relevant decisions under degraded connectivity; R6, independent physical confirmation of actuation; R7, machine-readable provenance and non-repudiable evidence; R8, secure lifecycle governance across onboarding, update, rotation and recovery and R9, bounded, predictable degradation under failure. R1–R4 and R8 are largely inherited from OT and IoT guidance; R5, R6 and R9 come from the irrigation-control and cyber-physical literature, where the physical process rather than the data is the object of protection; R7 comes from the provenance strand. R6 is not stated as a separate requirement in the general OT and IoT baselines reviewed, although closely related mechanisms exist in safety instrumented systems and process-aware detection (Section 1.1.3); in the form used here, it is specific to systems in which a command has an observable hydraulic consequence.

**Figure 4:**
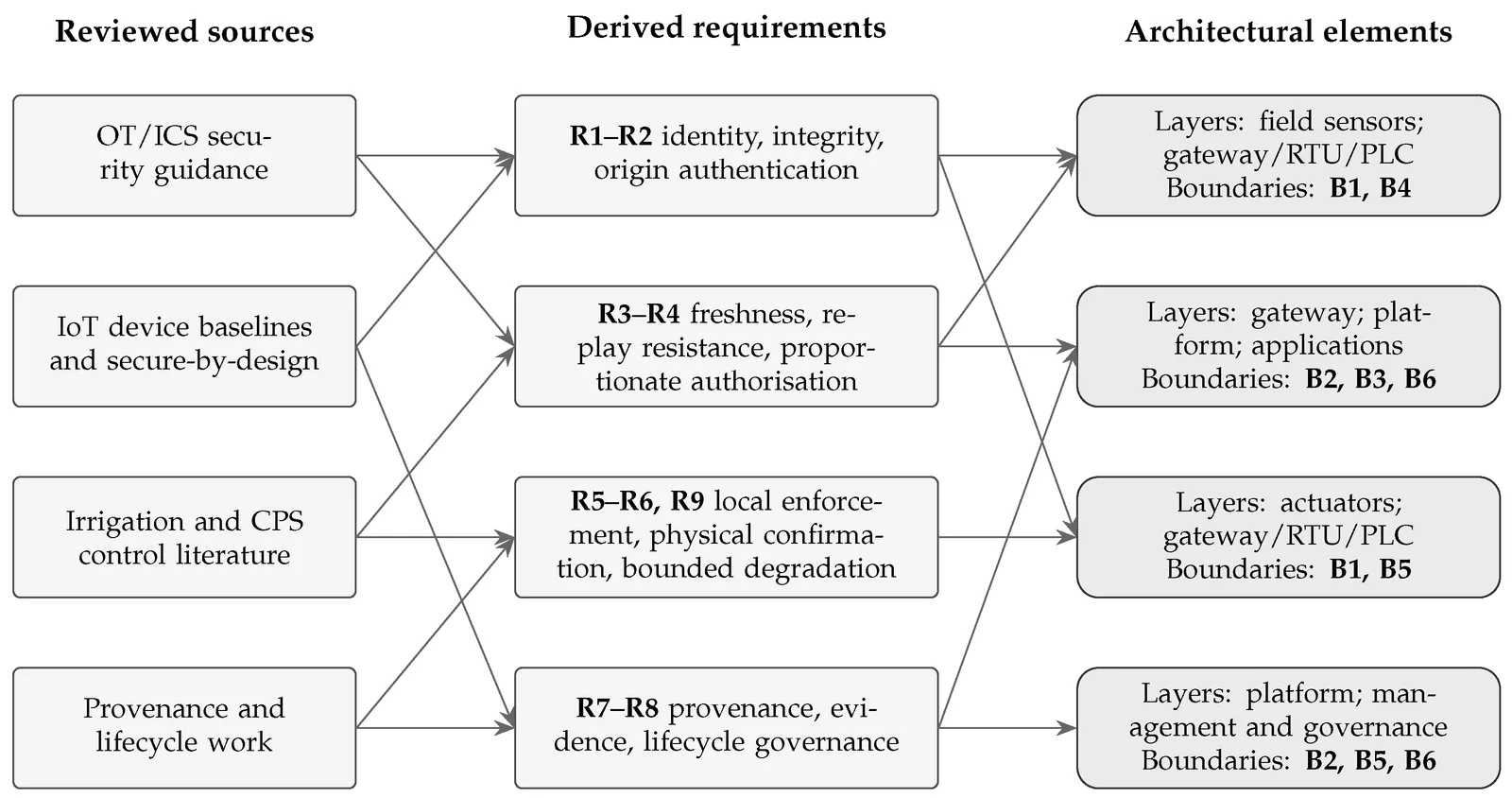
Derivation of the layered architecture and the B1–B6 trust-boundary classes from the reviewed security standards, security principles, and irrigation requirements. Each requirement group is discharged at one or more layers and re-established at one or more boundary crossings; the complete mapping is given in Table 6.

Figure 4 shows the derivation graphically, and Table 6 gives the full mapping from each requirement to its originating sources, the layers that implement it, and the boundary classes at which it must be re-established.

Reading Table 6 by column explains the form of the boundary classes. B1 appears against six of the nine requirements because the field–edge crossing is where a constrained, physically exposed device meets a component with authority over actuation, so the largest number of properties must be re-established there. B5 appears only against requirements whose assumptions can be invalidated physically rather than logically. B3 appears against a single requirement, R4, because the platform–application crossing changes *who* asserts authority rather than *what* is asserted. The six classes are in this sense the equivalence classes of crossings sharing a re-establishment obligation.

### 2.3. Reference Architecture

The proposed architecture is technology-neutral and can be mapped to LoRaWAN, Narrowband IoT (NB-IoT), Wi-Fi, Ethernet, Message Queuing Telemetry Transport (MQTT), the Constrained Application Protocol (CoAP), Open Platform Communications Unified Architecture (OPC UA), Modbus, Representational State Transfer (REST) application programming interfaces (APIs), or proprietary field protocols. The main architectural requirement is not the use of a particular technology, but the preservation of security and safety properties across layers and flows.

**Figure 5:**
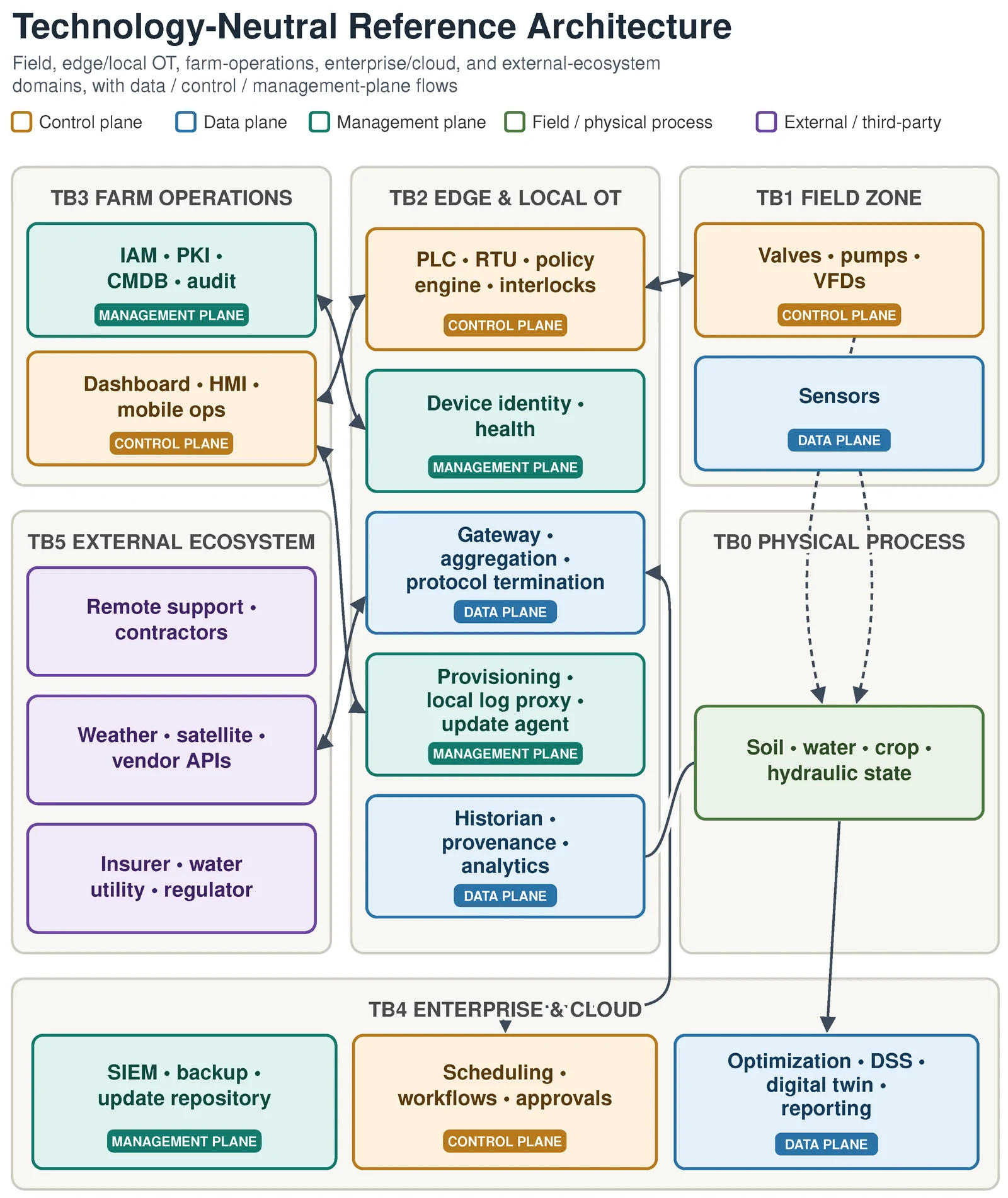
Technology-neutral reference architecture for trustworthy digital irrigation systems. The architecture separates field, edge/local OT, farm operations, enterprise/cloud, and external ecosystem domains, while distinguishing data, control, and management-plane flows. Each box is one function; its colour and tag give its plane (legend at top), and arrows show the principal flows only. Panel labels TB0–TB5 name architectural domains and are not the boundary classes B1–B6.

Figure 5 illustrates the architecture: the field zone holds sensors, actuators, local identities, and the physical process; the edge and local OT zone holds gateways, protocol mediation, policy engines, historians, update agents, and interlocks; farm operations cover dashboards, human–machine interface (HMI) and mobile functions, identity and key management, and audit; enterprise and cloud services provide orchestration, analytics, scheduling, reporting, backups, repositories, and decision support and the external ecosystem comprises vendors, contractors, weather and satellite feeds, insurers, water utilities, and regulators. Note that the panel labels TB0–TB5 identify *architectural domains*, a separate axis from the six *trust-boundary classes* B1–B6, which identify *crossings between* domains. The two are not in one-to-one correspondence, so the paper refers to panels by name and reserves B*n* for the boundary classes.

Table 10 summarises the functional layers and their minimum controls. The separation is logical rather than vendor-specific: a small farm may combine several functions in one gateway, a larger deployment distributes them. Orthogonal to the layers are three planes. The *data* plane carries measurements, alarms, state observations, derived features, metadata, and provenance; the *control* plane carries schedules, setpoints, override requests, policy and interlock decisions, acknowledgements, and physical feedback; the *management* plane carries onboarding, credentials, certificates, firmware updates, configuration changes, logs, backups, access policies, and incident response. The distinction matters because one asset participates in several planes. An edge gateway forwards telemetry, enforces valve commands, and receives signed firmware. Treating those flows identically creates hidden risk: a telemetry topic may tolerate delay, whereas a pump-stop command requires strict freshness, integrity, acknowledgement, and local execution guarantees, and a firmware update, though not time-critical, demands provenance, signature verification, rollback, and auditability.

### 2.4. Trust Boundaries and Enforcement Patterns

A trust boundary is any point where a datum, command, credential, configuration change, or evidence record crosses into a different set of assumptions about identity, authority, exposure, freshness, governance, or physical access. It is not limited to a firewall or network hop: reading from a broker topic, consuming an external feed, issuing a remote override, accepting a firmware update, or opening a controller cabinet are all trust-boundary events.

Table 2 defines the six boundary types, their transitions, the change in trust assumptions, the main risks, and the enforcement pattern required at each. The design question at every boundary is not whether communication is possible but whether the receiving component can trust the identity, freshness, integrity, and operational meaning of what it receives.

Every crossing should, therefore, re-enforce authentication, authorisation, integrity, freshness, auditability, and resilience in proportion to the risk borne by the receiving function. A dashboard command that opens a valve is not a user-interface event but a control-plane request entering the local OT environment; an external forecast is not merely information but a potential input to a decision that suppresses irrigation; a firmware update is not a file transfer but a modification of the controller’s future trustworthiness.

Security in smart irrigation is thus not reducible to encryption or network protection. Each boundary must establish who created a datum, command, or configuration change; whether it is fresh, unaltered, authorised and traceable; whether it can affect a physical action and whether enough evidence survives to audit the decision later. The controlling conclusion is that actuation authority remains with the local gateway, RTU, or PLC-like unit: cloud services, dashboards, and external feeds may request or recommend, but the local controller confirms identity, freshness, policy compliance, interlocks, and physical feedback before execution, so irrigation stays bounded and explainable when connectivity degrades, data goes stale, sessions are compromised, or components fail. The per-class obligations and the risks they answer are given in full in Table 2 and are not repeated in prose.

### 2.5. Irrigation-Centric Crown Assets

Asset-centric analysis is required because components differ in authority over the physical process: a sensor influences decisions, a controller converts decisions into commands, an actuator changes hydraulic state, a dashboard enables override, and a signing key can authorise code affecting every controller. Table 8 defines the six crown-asset classes, specifically those whose compromise directly affects irrigation safety, reliability, or physical function, with the trust level, safety impact, minimum controls, and typical misuse modes of each. The levels are analytical rather than certification levels: T0 untrusted or external inputs, T1 constrained field endpoints, T2 managed internal services, T3 control-mediating assets with authority over physical action, and T4 roots of trust such as the public key infrastructure (PKI), signing keys, and immutable audit stores.

To make the classification auditable rather than an unrecorded judgement call, each asset is assigned its level by an explicit decision tree supported by a scoring matrix. The tree asks in turn whether the asset is external or unmanaged (T0, unless additional provenance and verification controls exist); a constrained field endpoint that mainly produces data (T1); a managed internal service that processes, stores, or visualises information without authorising physical action (T2); able to mediate, authorise, execute, or block physical actions (T3); or the root of trust for other components (T4). The matrix scores five criteria: physical actuation authority, decision influence, credential or update authority, exposure to compromise, and consequence of failure (from 0 to 3), giving a total in [0,15], with anchors and thresholds in Table 7. The band is only a *default*, corrected by three rules. Rule E1: an asset scoring 3 on actuation authority is never below T3. Rule E2: an asset scoring 3 on credential, update, or trust-anchor authority is T4. Rule E3: since T4 is reserved for roots of trust, an asset scoring below 3 on credential authority is capped at T3 even when its aggregate falls in the T4 band. This resolves the case of a capable gateway that scores highly without becoming a trust anchor. The tree takes precedence over the score for constrained field endpoints, which stay in T1–T2 irrespective of exposure.

Because the rubric, the tree, and the three rules are applied in an order that matters, stating them only in prose leaves that order implicit. Figure 2 renders the whole procedure as one flow in the following order: entry tests, score, default band, then E2, E3 and E1. The figure is the normative statement of the procedure, and the branch taken is the entry recorded in the audit trail.

#### 2.5.1. Worked Example

Asset A1, a soil-moisture sensor, scores (0,2,0,3,2): no actuation, a primary but corroborated scheduling input, no credential, unmonitored outdoors, degraded reliability if spoofed. Its total of 7 defaults to T2, but the field-endpoint branch takes precedence and places it in the T1–T2 band. Asset A3, an RTU/PLC gateway, scores (3,3,2,2,3), a total of 13 falling in the T4 band by score alone; E3 caps it at T3 because it holds a long-lived service credential rather than trust-anchor authority, and E1 confirms it cannot fall below T3. Any code-signing sub-function it hosts scores 3 on credential authority, triggers E2, and is inventoried separately as an A6 asset at T4. Separating the cap from the escalation prevents a physically capable device from silently absorbing root-of-trust status, and the precedence order is itself recorded in the audit trail.

The rubric, therefore, makes the model checkable: a reader can verify how any level in Table 8 was reached, and the criteria can be reapplied across deployments. It remains structured expert judgement rather than a validated instrument, because no independent-rater agreement study has been conducted, and should be read as a checkable starting point for a deployment’s own risk assessment rather than a measurement of an asset’s “true” level. Establishing such reliability with external analysts is future work (Section 5.5).

#### 2.5.2. Robustness of the Classification Under Score Perturbation

Pending such a study, stability can be substantiated analytically. The concern behind a request for cross-verification is that a rubric applied by one team may be so sensitive to individual judgement that another analyst would reach a different level, and that sensitivity is computable from the rubric: perturb each criterion by one anchor step in each admissible direction and recompute the level under the tree and rules E1–E3.

Table 9 reports the result, with A4 split into its two sub-cases. Across all assets, 42 of 54 admissible single-step perturbations (78%) leave the level unchanged, and the pattern of the remaining 12 is more informative than the aggregate. A1 and A2 are completely insensitive, because E1 and the field-endpoint branch fix them independently of the score. A3 changes only upward, and only on acquiring credential-signing authority, precisely when it should be re-inventoried. A6 changes only if the criterion triggering its escalation is scored differently, a disagreement about what the asset *is*. The two genuinely sensitive cases, A4a and A5, sit on the T1/T2 and T2/T3 borders; this is a finding rather than a defect, since these are exactly the assets whose authority is indirect and, therefore, contestable. Two consequences follow: Table 8 reports A5 as a T2–T3 band, and adopters should treat a borderline case as a trigger for the escalation rules rather than for further argument about the score.

This measures the sensitivity of the classification function, not the agreement of analysts applying it. What it establishes is a bound an inter-rater study cannot invalidate for the safety-critical cases: for A1, A2 and A3 no single divergent judgement changes the resulting control obligations, and the residual uncertainty is confined to the two service classes now reported as bands.

**Figure 6:**
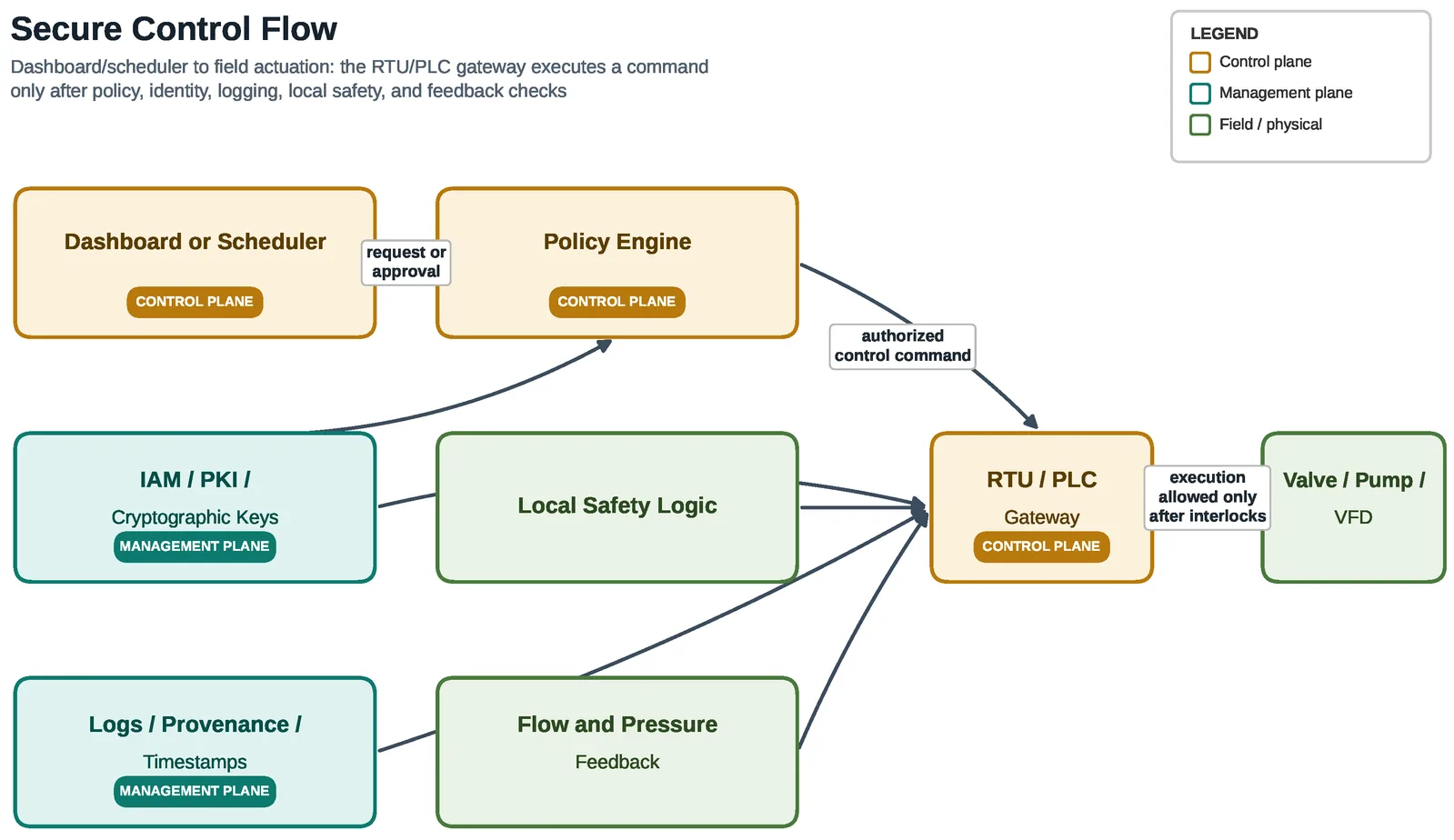
Secure control flow from dashboard or scheduler to field actuation. The RTU/PLC gateway executes a command only after policy, identity, logging, local safety logic, and feedback checks. The upper row is the request path (control plane), the left column supplies identity and evidence (management plane), and the flow and pressure feedback on the lower right is the physical confirmation that completes the command.

Figure 6 shows how these classes interact along one control path, making visible that the gateway rather than the requesting application converts a request into a physical action. The design conclusion is that controls may be implemented at different points, but the requirement never disappears: if a valve cannot support mutual authentication or signed commands, the controller driving it must enforce command verification, time limits, interlocks, and audit logging on its behalf; if a sensor cannot store strong credentials, the gateway compensates through onboarding controls, plausibility checks, sequence counters, physical inspection, and bounded decision logic.

### 2.6. Formal Control and Provenance Model

The trust levels of Section 2.5 determine *which* components need the strongest guarantees; this section specifies *how* they are enforced, through the two design principles and a machine-checkable telemetry-provenance model on which the threat analysis and the proof-of-concept both build.

- *Local Safety Authority:* The local gateway, RTU, or PLC-like controller should be the last logical enforcement point before actuation. Cloud services and dashboards may request actions, but local control logic must decide whether the request can be executed safely. This requires policy rules for permitted zones, maximum runtime, pressure limits, expected flow ranges, maintenance windows, emergency stops, and loss-of-context behaviour.
- *Feedback-Aware Execution:* Smart irrigation should be treated as a closed loop: perception, decision, execution, and feedback. A valve-open command should be followed by a corresponding physical signal, such as flow increase, pressure change, valve-state transition, or downstream soil-moisture response within a predefined time window. If command and feedback diverge, the controller should raise an alarm, prevent repeated unsafe commands, and move to a bounded safe state, whose choice between fail-closed and fail-open behaviour is itself a design decision (Section 2.6.4).

#### 2.6.1. Provenance and Secure Time

The system shall preserve machine-readable evidence sufficient to determine who or what produced each telemetry item, when, under which device configuration, whether it remains fresh, and which transformations preceded its influence on a decision. Provenance is a JavaScript Object Notation for Linked Data (JSON-LD) profile of the World Wide Web Consortium (W3C) PROV Ontology (PROV-O): an observation is a prov:Entity, the device a prov:Agent, the acquisition a prov:Activity, linked by prov:wasGeneratedBy, prov:wasAttributedTo and prov:wasDerivedFrom; calibration profiles, firmware images, and transformation algorithms may appear as further entities.

The minimum record carries eleven elements: a deployment-unique record identifier; the authenticated device identifier and zone; the observed property, value and unit; the measurement and trusted gateway-receive timestamps; a boot or session identifier with a monotonic sequence number; calibration and firmware versions; a controlled quality flag; the time-to-live; the clock source and estimated uncertainty; a payload digest or integrity proof and links to source records and transformation activities. A record *r* is eligible for autonomous control only when:

Accept(r)=Authenticated(r)∧IntegrityValid(r)∧Fresh(r)∧Ordered(r)∧QualityAcceptable(r)

Let $t_{m}$ denote the measurement time, $t_{g}$ the trusted gateway-receive time, $TTL_{r}$ the record time-to-live, and $u_{r}$ the declared or configured clock uncertainty. Freshness is defined as

$$-u_{r}\leq t_{g}-t_{m}\leq TTL_{r}+u_{r}.$$

A record is ordered when its sequence number is strictly greater than the last accepted sequence number for the same authenticated device and boot identifier:

$$seq_{r}>LastSeq\left(device_{r},boot_{r}\right).$$

The boot identifier prevents a legitimate sequence reset after restart from being mistaken for a replay. Duplicate identifiers, decreasing sequence numbers, expired TTLs, invalid integrity proofs, or timestamps outside the uncertainty window cause rejection or an INVALID marking. SUSPECT records may be stored for diagnostics but shall not independently trigger or suppress high-impact actions without local corroboration (Section 2.6.2). The controlled values of quality_flag are VALID, SUSPECT and INVALID; the clock source is drawn from a deployment-defined vocabulary such as GNSS (Global Navigation Satellite System), PTP (Precision Time Protocol), NTP (Network Time Protocol), RTC (real-time clock), GATEWAY_ASSIGNED or UNSYNCHRONISED. Where a device cannot keep trustworthy wall-clock time, ordering relies on the boot identifier and sequence number while the gateway assigns a trusted receive timestamp; the way that the time source, boot identifier and counters themselves are protected and recovered is specified in Section 2.6.3.

#### 2.6.2. Corroboration and Bounded Use of SUSPECT Records

Accept(r) is binary while the quality vocabulary has three states, and the relationship needs stating: a record can be structurally sound and cryptographically authentic yet carry a value the gateway has reason to distrust. Discarding such records is operationally unacceptable, since drift, partial occlusion, and post-rainfall transients produce readings that are genuine but individually unreliable, and treating every questionable reading as INVALID loses visibility exactly when irrigation decisions matter most.

**Figure 7:**
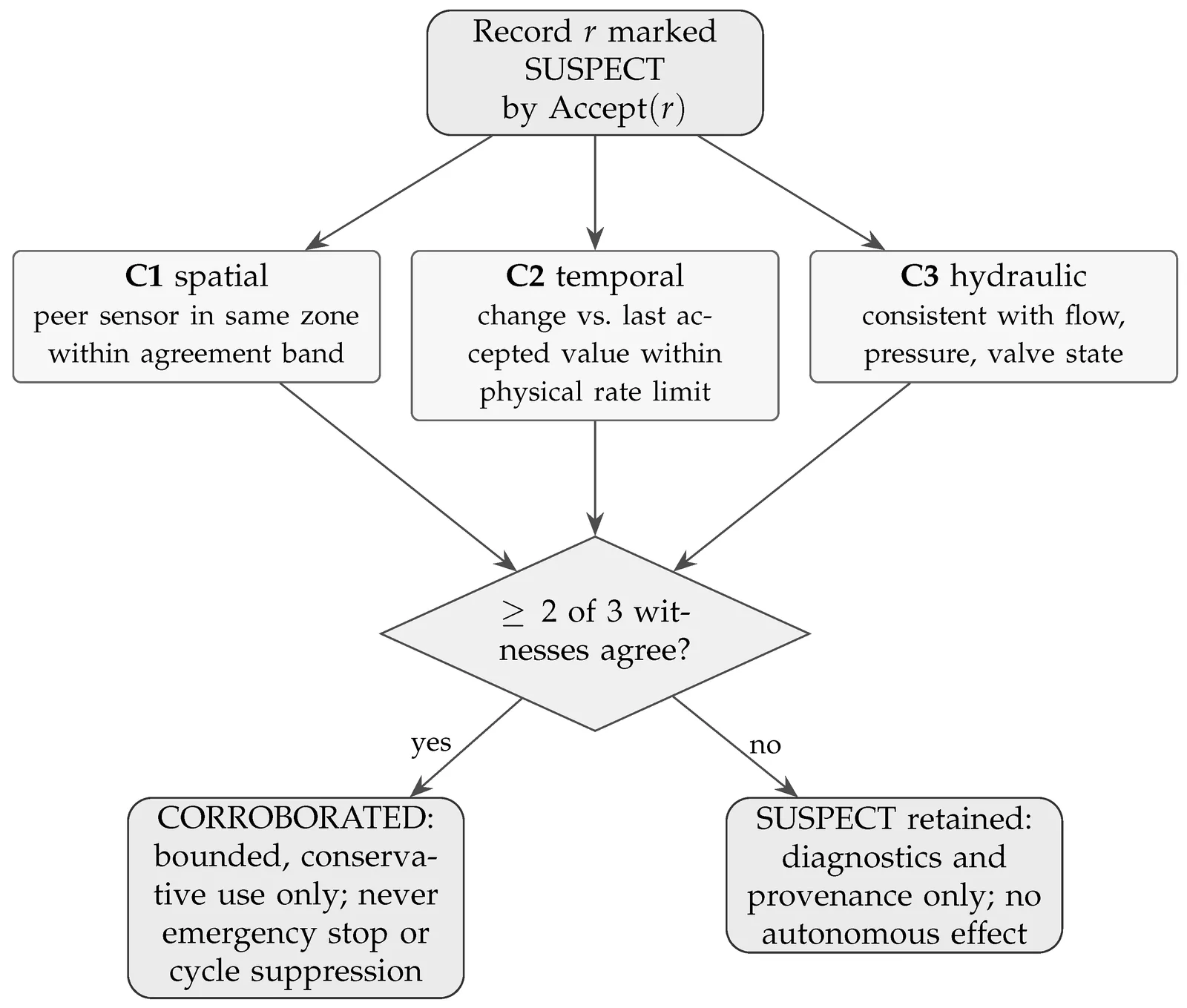
Corroboration procedure for SUSPECT telemetry. A record that is authentic, intact, fresh, and correctly ordered but implausible is quarantined rather than discarded, and may be promoted to CORROBORATED only when at least two of three independent witnesses agree; a promoted record remains restricted to bounded, conservative irrigation actions.

A record is, therefore, marked SUSPECT rather than INVALID when it passes authentication, integrity and ordering but fails a plausibility rule: the value lies outside the calibrated range, the rate of change exceeds the physical limit for the medium, the declared clock uncertainty exceeds its bound, or the device is under an open tamper indication. INVALID is reserved for failures of the record as an artefact, and only INVALID records are discarded without provenance. SUSPECT records are stored with a complete provenance entity and enter the corroboration procedure of Figure 7, which weighs three independent witnesses, chosen so no single compromised device can supply more than one:

∘ *C1, spatial.* A peer sensor observing the same property in the same zone reports a value within the configured agreement band over the same interval. The peer must be a distinct device with a distinct credential, so an attacker holding one key cannot manufacture agreement.
∘ *C2, temporal.* The value is consistent with the trajectory of the last accepted observations from the same device once the physical rate limit of the medium is applied, because soil moisture cannot fall faster than maximum evapotranspiration and drainage allow [70], so a step change is evidence of fault or attack rather than of dryness.
∘ *C3, hydraulic.* The value is consistent with control-plane state. A soil-moisture reading falling while the zone valve is confirmed open and flow is nominal is contradicted by the hydraulic evidence.

A SUSPECT record is promoted to CORROBORATED when at least two of the three witnesses agree, since two independent corroborators cannot come from one compromised endpoint. Promotion does not restore full trust: a corroborated record may contribute as one input among several to a scheduling decision and may trigger a *conservative* action whose failure mode is the safe one, such as continuing an approved cycle or raising an alarm, but it may not unilaterally initiate irrigation, extend a cycle beyond approved runtime, suppress a scheduled irrigation, or contribute to an emergency stop. The asymmetry is deliberate: corroboration lowers the probability that a value is wrong without eliminating it, so the permitted actions are those whose consequence stays bounded if it is. Below two witnesses, the record stays SUSPECT, available for diagnostics and audit with no autonomous effect; an operator may confirm it explicitly, and that confirmation is recorded as a B3 event with identity, reason code, and corroboration state.

This converts an otherwise discarded observation into evidence of defined and auditable weight. Had the injected value in the spoofed-telemetry scenario been authentic but implausible, as it would happen in the case of a genuine probe physically disturbed, it would have been marked SUSPECT rather than rejected, failed C1 and C3 against the peer sensors and hydraulic state, remained unusable for actuation, and surfaced as a maintenance alarm rather than a security event.

Any filtering, aggregation, unit conversion, imputation, or anomaly-detection operation shall create a new prov:Entity referencing its inputs through prov:wasDerivedFrom and its operation through prov:wasGeneratedBy, so raw observations are never overwritten and the transformation chain remains auditable. JSON-LD provides semantic interoperability but not integrity: the record shall travel over authenticated transport and should carry a signature or message-authentication code covering the canonical payload, provenance identifiers, timestamp, sequence number, boot identifier and TTL. Structural constraints may be validated with a Shapes Constraint Language (SHACL) shape, while freshness, monotonicity and signature verification are enforced at runtime by the gateway or ingestion service.

#### 2.6.3. Secure Time, Counter State, and Recovery After Disconnection

Considering the five conjuncts of Accept(r), only Fresh(r) depends directly on time, and QualityAcceptable(r) only indirectly, through the clock-uncertainty bound of Section 2.6.2; authentication, integrity and ordering do not, and command acceptance in Algorithm A2 depends on time only through the TTL check. The model’s dependence on freshness windows is, therefore, real but bounded: an adversary who corrupts the gateway clock can widen or narrow the window, and a widened window lets a record that was captured but never delivered be accepted late (a delay attack), whereas a replay of a record already delivered is still rejected by the sequence check. Five conditions nonetheless decide whether the freshness check means what it claims.

*Secure time sources.* The trusted gateway-receive time $t_{g}$ is the reference against which every record and command is judged, so the gateway clock is itself an input crossing B6. GNSS time is accurate but can be jammed or spoofed with inexpensive equipment, and unauthenticated NTP can be shifted by an on-path attacker. The gateway should, therefore, use authenticated time where available, such as Network Time Security for NTP [71], authenticated PTP profiles or authenticated GNSS services; cross-check at least two independent sources; keep a battery-backed RTC together with a monotonic counter that cannot be set backwards and refuse a clock step larger than a configured bound without operator confirmation. The age of a command is evaluated once, on receipt, against the disciplined gateway clock; thereafter its remaining lifetime is tracked on the monotonic clock, so a later wall-clock step cannot revive an expired command.

*Clock drift.* The uncertainty $u_{r}$ is not a constant: it grows with the time since the last trusted synchronisation, $u_{r}\left(t\right)=u_{0}+\rho \;\left(t-t_{\mathrm{sync}}\right)$, where ρ is the oscillator frequency tolerance. A 20 ppm crystal drifts by about 1.7 s per day, negligible against a 35-min telemetry TTL but not against a 30-s command TTL after a long isolation (about 52 s after 30 days). The gateway, therefore, tracks $u_{r}$ explicitly. Once it exceeds a configured bound, records whose freshness can no longer be decided are marked SUSPECT (Section 2.6.2) rather than VALID, and command freshness falls back from timestamps to a challenge–response scheme in which the command must echo a fresh gateway-issued nonce. Field devices without a trustworthy clock contribute no $t_{m}$ on which the gateway relies: their freshness is judged on the gateway-receive time and their ordering on boot identifier and sequence number (Section 2.6.1).

*Reboot behaviour.* Because the boot identifier separates sequence epochs, its generation is security-relevant: it must never repeat for a given device. It is either a monotonic boot counter in non-volatile memory, incremented before the first transmission after power-up, or a random value of at least 64 bits. The gateway accepts a new epoch only from an authenticated record and retires superseded epochs, rejecting any boot counter lower than the highest seen or, for random identifiers, any identifier in the device’s retired set. Without this rule, an attacker could replay records from an old epoch whose sequence numbers would appear new against an unseen boot identifier, and for clock-less devices freshness would not catch the replay, because the gateway itself assigns the receive time.

*Counter loss.* If a device loses its transmit counter (brownout, flash wear), it must open a new epoch, never restart the counter within the old one. To avoid frequent flash writes, the device may lease counter values: every *N* increments it stores in non-volatile memory the value *N* ahead of the current counter, and after a restart resumes from the stored value, so sequence numbers may be skipped but are never reused. The gateway’s receive-side registers (LastSeq, lastCmdSeq, seenIds) must not be underestimated after a restart, since that would reopen a replay window; they are, therefore, written through to non-volatile storage before the corresponding record or command takes effect. If that state is nevertheless lost, the gateway enters a re-synchronisation mode in which telemetry is marked SUSPECT and commands are refused until each device has opened a new epoch in response to a gateway challenge. Command replay memory is bounded by the command TTL: seenIds need to retain only identifiers younger than the TTL plus clock uncertainty, which keeps that state small enough to persist on every update.

*Recovery after prolonged disconnection.* After a field–edge outage, devices that buffered records deliver them late; they fail Fresh(r) and so cannot drive control. They remain valuable as history, so the architecture permits a separate archival path on which authenticated, correctly ordered but stale records are ingested into the historian, explicitly marked as non-control data, and never admitted to Accept(r) or to the corroboration witnesses for current decisions; Algorithm A1 specifies the control path only. After an edge–platform (WAN) outage, the gateway has operated under bounded offline autonomy, and commands queued upstream during the outage have expired by TTL; they are not executed as a backlog on reconnection, and the scheduler must reissue them against the current state. Before resuming normal autonomous operation, the gateway runs a logged recovery sequence: re-establish trusted time and reset $u_{r}$; refresh credential-revocation status, since a revocation issued during the outage would otherwise be ignored; reconcile counters and epochs and confirm the current hydraulic state through feedback before accepting new high-impact commands.

The proof-of-concept of Section 4.2 exercises freshness only through fixed record and command ages (scenarios S2 and S5); clock drift, time-source spoofing, counter loss and epoch replay are not injected and are listed as future work in Section 5.5.

#### 2.6.4. Selecting the Bounded Safe State: Fail-Closed Versus Fail-Open

Algorithm A2 reverses an unconfirmed valve opening into a closed valve, and Table 11 lists closing valves and stopping the pump as the immediate irrigation response. That default is a choice rather than a law: neither fail-closed nor fail-open behaviour is safe in every context, and each fails in a way the other avoids. Table 12 summarises the trade-off.

*Fail-closed* behaviour (close valves, stop pumps, hold) protects equipment and the water budget: it prevents dry running when a reservoir or well empties, overpressure and water hammer, pipe rupture, flooding or erosion from a stuck-open valve, and over-application in fertigation. It harms the crop when it persists. An extended hold during a heat wave, at flowering or fruit set, or on shallow-rooted or container-grown crops with little stored soil water can cause irreversible yield loss within hours to days, and in saline soils a missed leaching irrigation allows salts to accumulate in the root zone. Fail-closed behaviour is also an attack surface: an adversary who can block feedback, jam the field link or trigger tamper alarms can turn the safety mechanism into a denial-of-irrigation attack, achieving through the controller the suppressed irrigation that would otherwise require a spoofed high soil-moisture reading (Section 1).

*Fail-open* behaviour (continue the last approved schedule, or leave a supply valve open) preserves the crop and, for livestock watering, animal welfare, but wastes water, may breach a volumetric allocation or abstraction licence, waterlogs soil, leaches nitrate below the root zone and generates runoff and erosion. Where it is triggered by lost feedback, it also removes the evidence that the hydraulic system is within limits: a burst lateral or stuck-open valve continues unobserved, and a pump may run against a closed downstream valve or an empty supply.

The architecture, therefore, does not fix a single safe state. It requires a graded, time-bounded safe state per zone, specified at commissioning (step 3 of Section 4.4) and recorded in the local policy. (i) On loss of feedback or of a trusted input, the affected zone, not the whole farm, holds immediately. (ii) If the condition persists beyond a crop- and season-dependent deficit-tolerance window, the zone reverts to a conservative, pre-approved fallback programme, consisting of shorter, lower-volume cycles derived from the last trusted schedule and local evapotranspiration climatology, executed under interlocks that do not depend on the failed channel: a pressure switch, pump-current or run-dry protection, a maximum runtime, and a maximum volume from the flow-meter totaliser. (iii) The operator is asked to confirm or adjust, and that confirmation is recorded as a B3 event. Protective functions such as pressure relief and run-dry or overpressure trips remain unconditional throughout and are never overridden by the fallback programme (Section 4.3). The feedback-timeout response verified in Section 4.2 implements stage (i) only; a deployment that stops at stage (i) has chosen equipment protection over crop protection, and should record that as an explicit risk acceptance. Livestock watering, where availability is itself the safety property, reverses the default and starts from a bounded fail-open state (Table 11).

### 2.7. Sensor-Level Integration: From Physical Sensing Characteristics to Trust-Boundary Controls

The model so far treats a telemetry record as an object with cryptographic and temporal properties. That is necessary but insufficient, because the record comes from a physical transducer whose behaviour determines what a plausible value even is: a freshness window is meaningless without a sampling interval, a plausibility check unimplementable without a calibrated range and drift model, and the attack-versus-fault distinction, which decides whether the response is a security alert or a maintenance ticket, is decidable only against specific hardware fault modes. This section states how hardware parameterises the B1 boundary.

#### 2.7.1. Sampling Rate, Freshness, and Duty Cycle

The freshness bound $TTL_{r}$ cannot be chosen independently of the acquisition schedule: a probe sampled every 15 min over a duty-cycle-limited link cannot support a 60-s TTL, and setting one guarantees legitimate records are rejected. The architecture requires $TTL_{r}\geq k\cdot \Delta t_{s}+u_{r}+t_{\mathrm{tx}}$, with $\Delta t_{s}$ the sampling interval, $t_{\mathrm{tx}}$ the worst-case transmission and queuing delay, $u_{r}$ the clock uncertainty, and k≥1 a tolerance for missed transmissions. The converse also binds: an actuation policy must not rest on a state estimate older than the process time constant it controls, so the feedback window of Algorithm A2 is bounded below by the hydraulic transport delay of the zone and a soil-moisture-driven decision bounded above by the drainage time constant of the soil. A slow-sampled probe gating a fast actuator is incompatible with that requirement, because the fast loop must close on flow and pressure, observable at the gateway, and the slow sensor must be admitted only as a scheduling input. This is a hardware-driven constraint on where a measurement may appear in the control chain, invisible from a protocol-level view.

#### 2.7.2. Measurement Drift and the Attack/Fault Ambiguity

Drift complicates enforcement most directly, because slow drift and a patient attacker look alike within a single record. Capacitive probes drift with electrode corrosion and post-fertigation conductivity changes, pressure transducers show thermal zero-drift, and low-cost devices show both more strongly and with wider unit-to-unit variation. The architecture separates the two questions: Accept(r) decides whether the record is what it claims to be; the corroboration machinery of Section 2.6.2 decides whether its value can be believed. Drift then appears as a rising rate of SUSPECT classifications from one device, correlated across time but *not* across the spatial witness C1, whereas a spoofing campaign typically fails C3 from its first record. Because the provenance record carries calibration version and device identity, the SUSPECT rate per calibration epoch is computable, and a rising rate is an actionable maintenance signal: the calibration metadata mandated at layer one is not documentation but the state variable that makes drift separable from attack.

A field deployment would surface several such transients that this analytical treatment cannot itself exercise, and each maps onto the machinery above rather than around it. First, a soil-moisture drop exceeding the evapotranspiration-based rate bound of C2 can be genuine rather than an artefact. Field measurements show that, under ordinary conditions, root-zone soil moisture declines at a rate set by evapotranspiration demand, which is what makes such a bound a useful plausibility test [70]. Preferential or macropore flow, however, through cracks, root channels and biopores, bypasses the soil matrix and can drain water far faster than a matrix-flow (Darcian) estimate predicts [72]. The C2 rate limit must, therefore, be conditioned on soil structure and recent-irrigation state rather than fixed, or such episodes will be misclassified as implausible.

Second, a wetting front passing the probe depth after irrigation or rainfall produces a rapid but physically genuine rise, distinguishable from a spoofed value mainly by its consistency with the infiltration timeline (C2) and with neighbouring probes at a similar depth (C1).

Third, capacitive probes infer moisture from the bulk dielectric permittivity of the soil, which is dominated by the permittivity of water [73], whereas resistive probes infer it from the electrical resistance of the soil; both readings also depend on soil properties such as particle size [74]. The measured permittivity is temperature-dependent [75], as is the electrical conductivity of the soil solution on which resistive readings rely, so diurnal temperature swings impose an apparent moisture cycle unrelated to water content. Because this bias is periodic and correlates with the co-located temperature channel rather than with an attacker’s objective, it calls for a temperature-compensated plausibility band, already required for the pressure-transducer class in Table 13, rather than treatment as a security event.

Fourth, shrink-swell cycling in clay-rich soils opens or closes the air gap at the probe–soil interface already listed for this sensor class in Table 13, producing a step change with no corresponding change in true moisture. None of these is distinguished from an attack by Accept(r), which governs the record’s authenticity rather than its plausibility; all four are exactly the province of the corroboration witnesses C1–C3 and the SUSPECT state of Section 2.6.2, and their concrete implication is that the plausibility bounds should be structure- and season-aware rather than the single fixed rate limit stated here. Calibrating C1–C3 thresholds against these specific phenomena is field work this paper does not perform, and it is added accordingly to the immediate next steps of Section 5.5.

#### 2.7.3. Fault Modes and Hardware-Tiered Verification

Physical fault modes must be enumerated per sensor class, because each produces a distinct signature and demands a distinct edge response; Table 13 gives the mapping for the classes typical of an irrigation deployment. Two architectural consequences follow.

First, the verification burden at B1 is inversely proportional to hardware quality. A premium transducer with an internal reference, calibration certificate and on-board diagnostics discharges part of R2 and much of the plausibility obligation itself and can often carry a secure element discharging R1. A low-cost probe, which is increasingly the rational choice at the densities that make precision irrigation worthwhile, discharges none, and every obligation migrates to the gateway. This is the compensation principle of Section 2.5: the requirement does not disappear; it relocates. The architecture, therefore, defines two hardware tiers at B1, labelled H1 and H2, to keep them distinct from the methodology steps S1–S6 and the scenarios S1–S7. Tier H1 devices support per-record authentication, monotonic sequence numbering and calibration reporting and Accept(r) is evaluated as specified. Tier H2 devices support some or none of these, so the gateway supplies the missing properties, such as trusted receive timestamp, sequence state, a plausibility band derived from datasheet accuracy rather than process physics, and spatial corroboration by default rather than only on failure. Tier H2 devices are consequently never sole inputs to an actuation decision, an architectural constraint rather than a recommendation.

Second, the tiering has a cost consequence quantified in Section 4.3: the redundancy that makes Tier H2 devices usable is what pays for their lower unit cost, and a deployment adopting low-cost sensing without the corresponding corroboration density has not saved money but silently lowered its assurance level. Uncertainty propagation across the layers is bounded not by any single device’s accuracy but by the corroboration structure imposed at B1.

## 3. Threat Model and Lifecycle Governance

This section addresses risk at two time scales: Section 3.1 gives a STRIDE-based point-in-time view of the attack surface, and Section 3.2 considers risk over the device and software lifecycle.

### 3.1. Threat Model and Risk-Oriented Mapping

The threat model addresses attacks and failures affecting irrigation safety, reliability and auditability, such as malicious action, field failure, operator error, stale data and compromised management-plane functions, interpreting STRIDE consequences in irrigation-specific terms. Documented work confirms such attacks are not hypothetical, covering unauthorised watering, replay, spoofing, wireless disruption, sensor faults, and attacks on smart-farm infrastructure [6,7,8,9], affecting the availability and correctness of physical processes rather than only of data.

Table 4 crosses every crown-asset class with every boundary class, recording for each of the 36 cells the dominant STRIDE categories and a qualitative impact rating. It is an architecture-level instrument for comparing how asset classes are exposed at specific crossings. The rating is a prioritisation measure: Low denotes limited operational effect, Medium degraded reliability or auditability, High potential unsafe irrigation, equipment stress or loss of control integrity, and Critical compromise of local actuation authority, root-of-trust material or system-wide governance. These 36 ratings are analytical judgements made against those anchors rather than measured or independently cross-validated values, and adopters should re-derive them against their own inventory and risk tolerance.

Six cells rate Critical, and they cluster rather than spread. The first cluster is the local controller at its edge–platform and physical-tamper crossings (A3 × B2, A3 × B5), where queue poisoning, stale commands, debug-port abuse, secret extraction or firmware manipulation subvert local actuation authority itself rather than merely misinform it. The second is identities and credentials at four of six crossings (A6 × B2, B3, B5, B6), where compromised service identities, stolen tokens, extracted secrets or abused supplier accounts undermine every other control at once. That concentration is the design message: the two classes most needing protection are the local enforcement point and the trust anchors authenticating it, specifically the pairing on which local safety authority depends. Elsewhere, boundaries closest to the physical process (B1, B5) dominate for telemetry and actuators, while those closest to human and organisational authority (B3, B6) dominate for dashboards and credentials.

### 3.2. Lifecycle Governance and Interoperability

The operational path runs from field sensor and gateway to edge controller or PLC and finally the valve, and must be protected across the whole device and software lifecycle. Onboarding, certificate issuance, credential provisioning, key rotation, firmware and configuration distribution, vulnerability handling, supplier access, backup and recovery are architectural functions rather than administration performed outside the system: a controller of secure original design becomes unsafe if it runs outdated firmware, reuses credentials, accepts unsigned configuration, or permits undocumented vendor access.

The architecture is protocol-independent: sensors may use LoRaWAN or another low-power technology, MQTT may link gateway to remote services, OPC UA may integrate industrial systems, Hypertext Transfer Protocol Secure (HTTPS) APIs may serve dashboards, and Modbus may persist inside a segmented OT network. What matters is not uniformity but preservation of identity, integrity, freshness, authorisation, accountability, auditability and safe failure behaviour across every transition from sensor to gateway to controller to valve. This follows connectivity studies showing no single stack fits every part of a deployment, specifically LoRaWAN and NB-IoT suit low-power remote telemetry, MQTT, OPC UA, REST middleware and fieldbus gateways serve higher-level integration [76,77,78,79], and must account for technology-specific exposure, since LoRaWAN carries physical- and network-layer attacks of its own [80,81] and legacy Modbus requires isolation [82,83].

Detection and response close the loop. Security-relevant events from the gateway, edge controller and PLC reach the security information and event management (SIEM) or intrusion-detection system, which alerts the operator to suspicious behaviour, unauthorised access or abnormal commands; depending on severity, the operator may apply a manual valve override, isolate the affected segment, or restore configuration from a trusted backup, so detection connects to containment and recovery rather than functioning as passive monitoring [84].

These controls are not free, and three cost dimensions deserve mention even without a hardware campaign. *Latency*: feedback-aware execution withholds success until the feedback window elapses, and signature verification and sequence bookkeeping add further delay; negligible against hydraulic response times for loops operating over seconds to minutes, but to be verified for fast-acting functions such as pressure relief or emergency shutoff. *Computation and storage*: signature verification, sequence tracking and minting a PROV-O record per accepted item impose recurring central processing unit (CPU), memory and flash costs at the gateway, entirely so for endpoints that cannot sign for themselves, and this cost must be provisioned for as device density rises. *Bandwidth*: ingest logging and provenance metadata add payload beyond the raw value, a materially larger fraction over a duty-cycle-limited low-power wide-area network (LPWAN) link, so such deployments may batch or locally digest provenance. None of this invalidates the architecture, but it argues against a uniform “full baseline” and for progressive adoption.

Deployments differ in funding, expertise, connectivity, environment and device capability. A minimum baseline comprises asset inventory, network segmentation, unique device identities, controlled remote access, local operational limits, manual override, backups and basic logging. Mature deployments extend this with automated PKI and certificate lifecycle, remote attestation [69], signed firmware and secure over-the-air update [68,85,86], redundant links, anomaly detection, software bill of materials (SBOM) management [87], provenance, secure supply-chain practices [88,89], and formal incident-response and disclosure exercises [90].

## 4. Results

Next, we present the evaluation design: six attack and failure scenarios plus one nominal-operation specificity check, each with an expected response and the evidence required for a pass/fail decision. It is placed here because the acceptance criteria it defines are the measurement instrument for every subsequent result. Section 4.2, Section 4.3, Section 4.4 and Section 4.5 then report the proof-of-concept outcomes, the quantified overhead, an illustrative worked deployment example, and the transfer to adjacent settings.

### 4.1. Verification Framework

**Figure 8:**
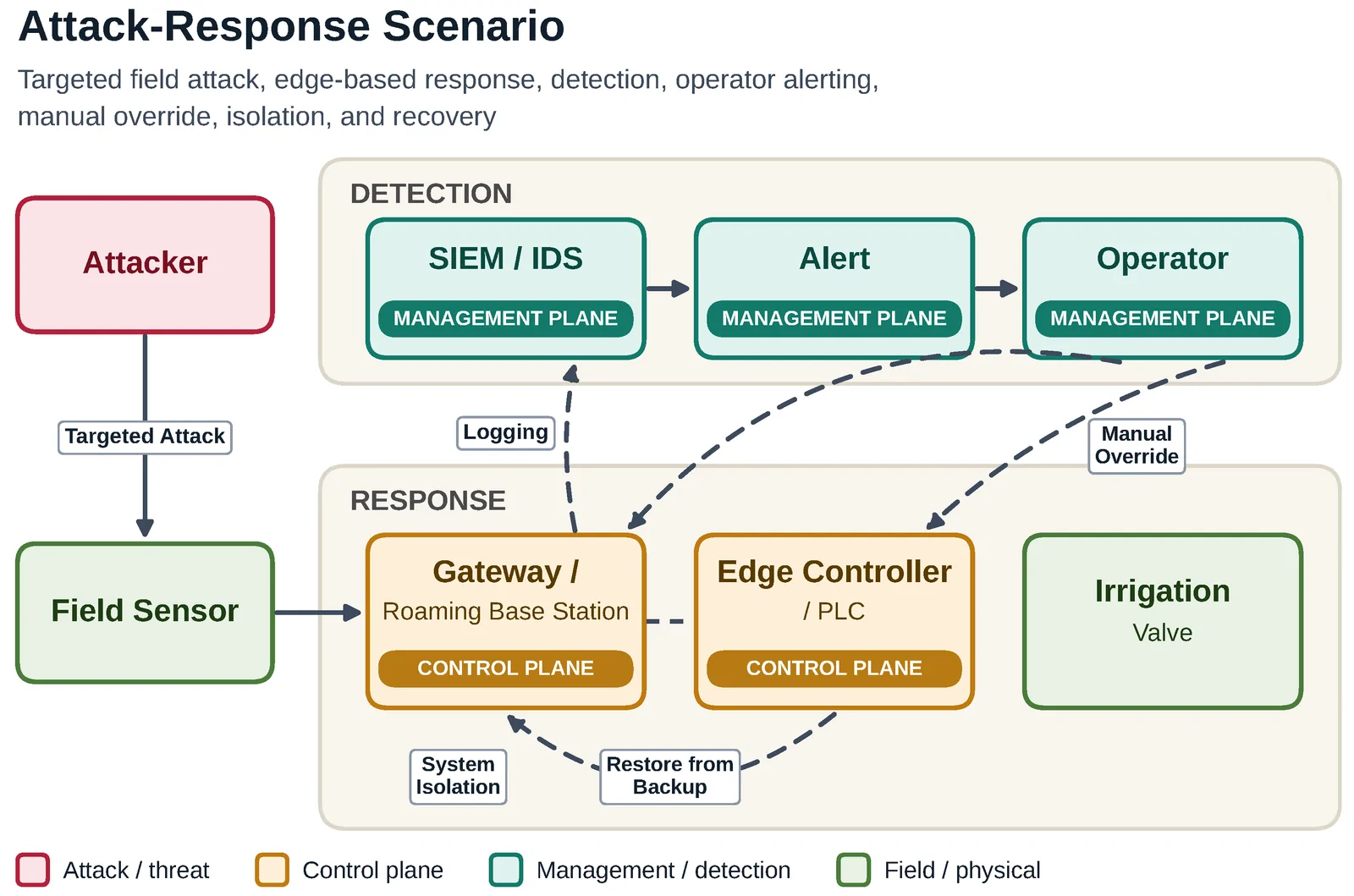
Illustrative attack–response scenario showing a targeted field attack, edge-based response, detection, operator alerting, manual override, system isolation, and recovery flows. Solid arrows are the attack and data path from left to right; dashed arrows are response actions (logging, manual override, isolation, restore from backup).

Since the model is a reference architecture rather than a fixed implementation, verification inquires whether a concrete deployment preserves its assumptions. Each scenario injects a defined attack, component failure, communication disruption or unsafe command and observes the whole control chain. Figure 8 shows one end-to-end, from injection through edge response, detection, operator alerting, manual override, isolation and recovery. Evaluation verifies that the event is detected, that an alert reaches the operator, that local safety limits hold, that override, isolation, or restoration succeeds, and that sufficient evidence is recorded at every stage, in detail: affected component, origin and time, commands transmitted, operator response, final valve state, so logging supports investigation, accountability and recovery verification as well as real-time detection.

The six attack scenarios were chosen not for prominence in the threat literature but by construction from the model, so the set is minimal and complete with respect to the architecture’s own claims. The rule was the smallest set of injections such that every boundary class is exercised, every crown-asset class appears as a target or protected asset, every enforcement mechanism is the *sole* control standing between injection and unsafe outcome, and the dominant STRIDE categories of the corresponding matrix cells are covered. The third criterion is the demanding one: each scenario tests exactly one mechanism, so a failure identifies which architectural claim is false rather than merely that something went wrong. Table 14 records the derivation. The set covers all six boundary classes and the categories S, T, R, D and E; information disclosure is deliberately not primary in any scenario, because confidentiality of irrigation telemetry, while it has economic and privacy consequences addressed on the management plane, cannot by itself cause an unsafe hydraulic action.

Construction from the model guarantees internal coverage but not representativeness, so the set was cross-checked against documented incident classes. S1 and S3 correspond to failure modes reported for smart-farm testbeds under wireless spoofing and deauthentication [7]; S2 is the mechanism enabling coordinated valve actuation in the irrigation-botnet analysis [6], replay protection being absent by design on commodity nodes [8]; S4 and S6 correspond to the credential-abuse and supply-chain categories dominating the systematic review [9]; S5 covers the third-party dependency arising from external weather and satellite services. Two limits: the set is not frequency-weighted, since the architecture claims nothing about relative likelihood, only about which control must hold where and it is exhaustive only with respect to the enforcement mechanisms introduced here, which is the property that makes a failure diagnostic. A deployment facing a threat outside the set should extend the table using the same criteria.

The asymmetry between six injections and one nominal check is deliberate. Attack scenarios each isolate a different mechanism and so contribute independent information about sensitivity, whereas specificity is a property of the whole enforcement path: the nominal scenario exercises it end to end by submitting a legitimate instance of every gated input type, specifically telemetry, command, override, feed, firmware update, in one run, testing five acceptance decisions rather than one.

Table 5 operationalises this by specifying, for each scenario, the injection method, the expected architectural response, and the evidence required to reach a reproducible pass/fail decision, so covering device identity, message freshness, command integrity, physical process safety, privileged-access control, external-data reliability, and update authenticity.

**Figure 9:**
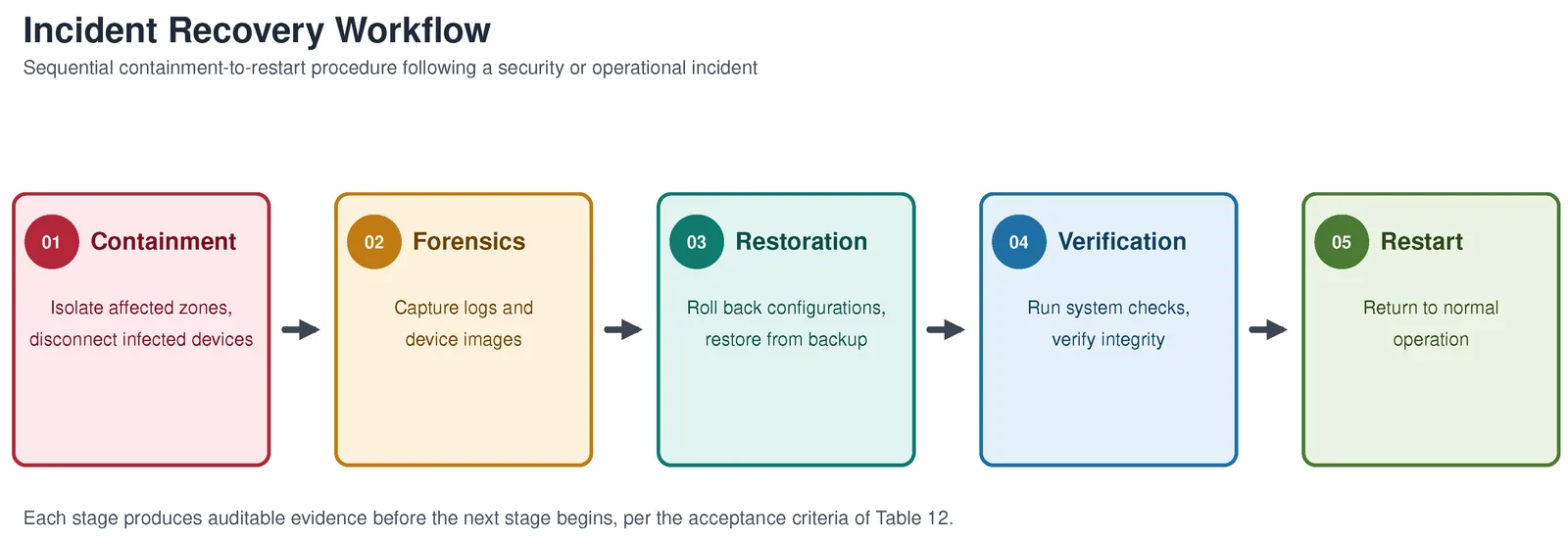
Illustrative recovery workflow showing containment, forensic evidence collection, restoration, verification, and controlled restart following a security or operational incident.

A deployment is compliant only when unsafe actuation is prevented or bounded, detection and response activate, the system reaches a defined safe state, and the specific evidence named in the final column of Table 5 is preserved. The framework thus assesses not whether individual controls exist but whether they operate coherently from field sensing through gateway processing, edge control, actuation, operator intervention and recovery, which makes it usable for procurement, design review, test planning and future testbeds. Following containment, the architecture supports the recovery workflow of Figure 9: isolate affected zones and devices, preserve logs and device images for forensics, restore trusted configuration from backup, and validate integrity and operational safety before a controlled return to normal operation.

### 4.2. Software-Based Proof-of-Concept Verification

A small proof-of-concept (PoC) program was written in Python to test the claims of Section 2.4 and Section 4.1, in the verification sense defined in Section 2.1. The PoC does not model any vendor product or communication protocol. It models only the two parts that decide the outcome of each scenario: the enforcement logic of the field gateway/RTU/PLC, and the physical process that this logic controls.

**Figure 10:**
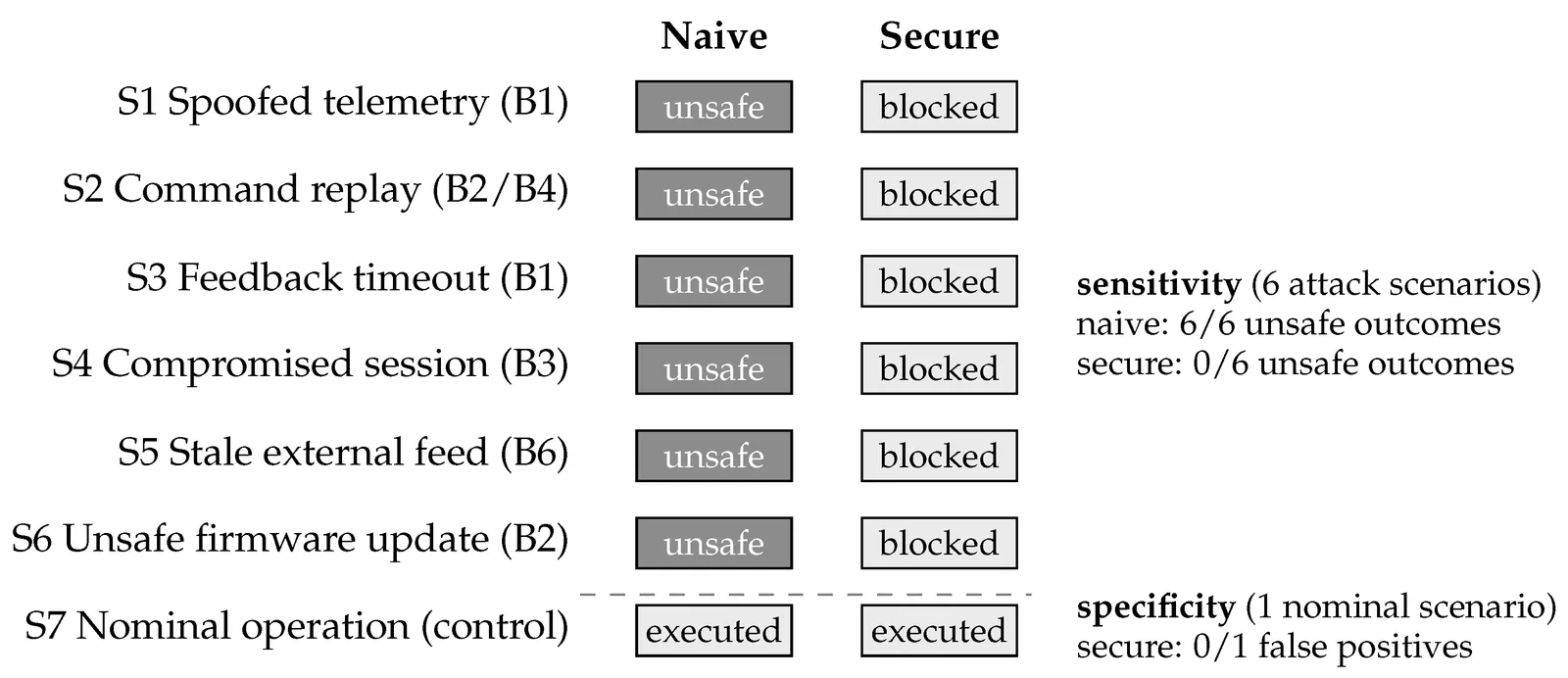
Proof-of-concept outcomes. Shaded cells denote an unsafe outcome; the dashed rule separates the six attack scenarios from the nominal-operation control. The secure controller blocks every attack scenario while executing the legitimate operations, so the separation is not produced by indiscriminate rejection. The column labelled “Naive” is the intentionally unsecured negative control.

Two controllers were built on the same process model. The first is an *intentionally unsecured negative control*: it has no boundary enforcement and accepts all telemetry, commands, feeds, overrides and updates as they arrive. It is not a baseline representative of existing irrigation systems, many of which implement some of these checks. Its only role is to confirm that each injected scenario is capable of producing an unsafe outcome when the corresponding control is absent, so that a blocked outcome under the secure controller can be attributed to that control rather than to an ineffective injection. Documented weaknesses in commodity sensor nodes [8], irrigation controllers [6] and schedulers [7] show that such a configuration is not hypothetical, but they are not used here to claim that it is typical. For brevity, it is labelled “Naive” in the code and in Figure 10. The *secure controller* implements the proposed mechanisms: the Accept(r) predicate, local safety authority and feedback-aware execution, freshness and provenance checks at B6, multi-factor authentication (MFA) and context-bound approval at B3, and verification that firmware is signed and belongs to an approved rollout. Both controllers act on the same simulated valve (a shared Valve object). Opening the valve produces an independent flow reading unless the attacker blocks that reading, so the feedback-timeout scenario tests a physical effect and not only a software check.

**Figure 11:**
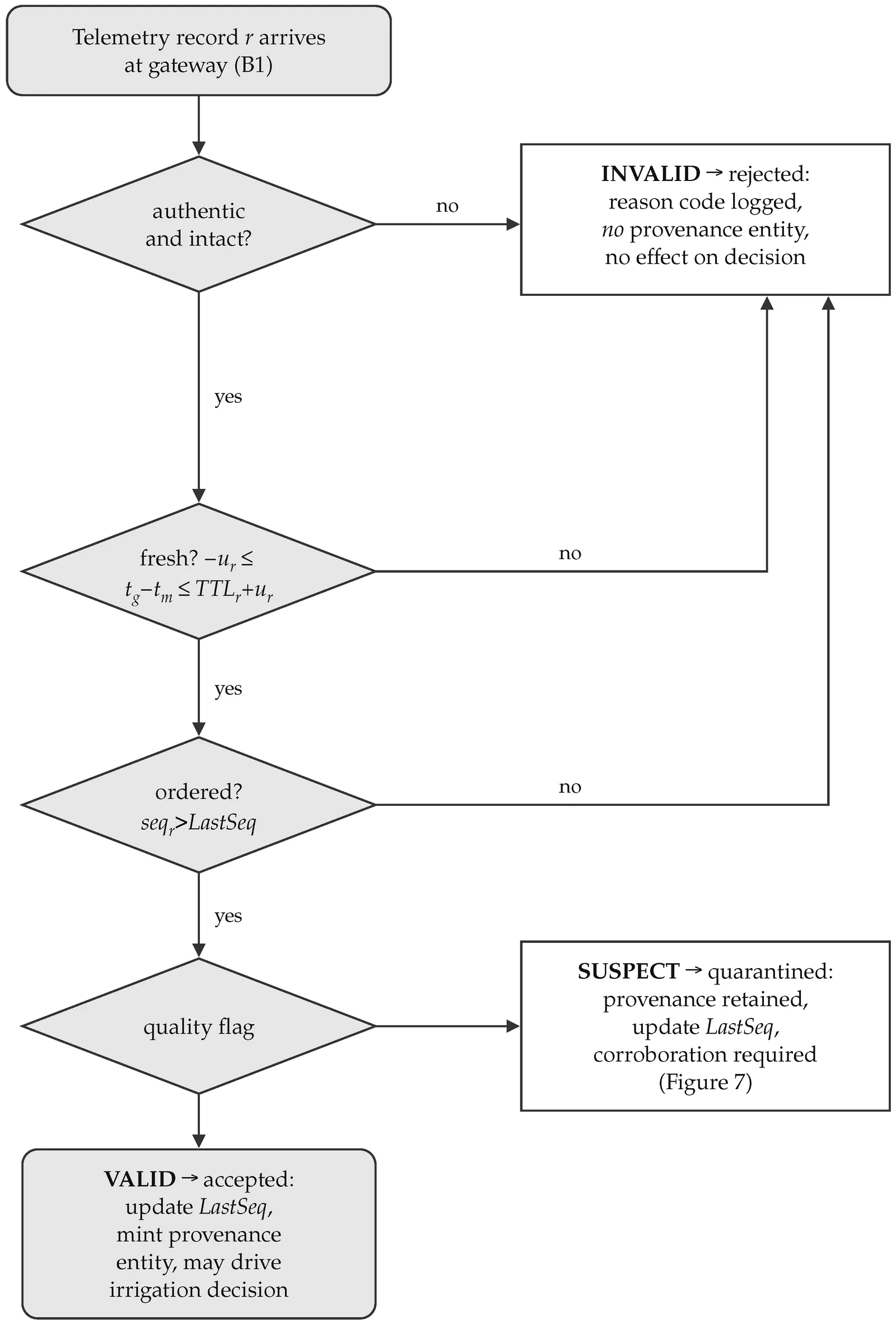
Data-plane enforcement point: telemetry acceptance Accept(r) at the field–edge boundary B1. State is mutated only after authentication, freshness and ordering have all passed (on both the Valid and Suspect branches), so a rejected (Invalid) record cannot advance the replay window. Pseudocode in Appendix B, Algorithm A1.

**Figure 12:**
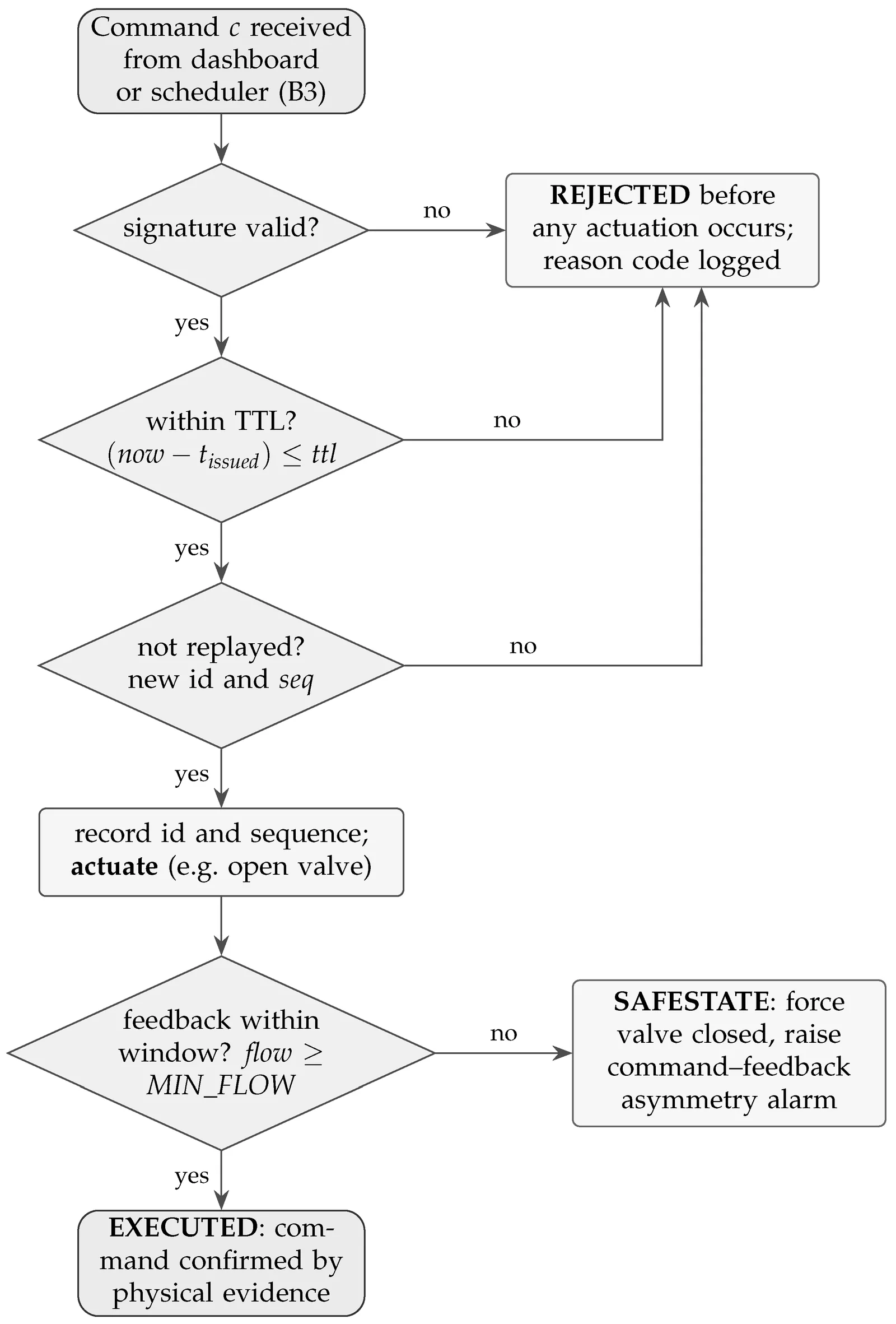
Control-plane enforcement point: local safety authority with feedback verification. Authorisation failures are refused before actuation; a physically unconfirmed action is reversed into a bounded safe state, since the absence of expected feedback is observable only after the command is attempted. Pseudocode in Appendix B, Algorithm A2.

The enforcement points appear as decision flows in Figure 11 and Figure 12: the data-plane point implementing Accept(r) and routing outcomes to acceptance, quarantine or rejection, and the control-plane point implementing local safety authority, gating actuation on signature, TTL, replay and sequence, and withholding a successful status until physical feedback confirms the action. The pseudocode the PoC implements line for line is Algorithms A1 and A2. Implementing them line for line also tests whether the prose specification is executable as written, surfacing ambiguities a textual description can hide. Two properties are what the scenarios test. Rejection precedes any state mutation: the last-sequence register advances only after authentication, freshness and ordering have all passed, for both accepted and suspect records, so a rejected (Invalid) record cannot advance the replay window and suppress a later legitimate one. And the feedback check sits *after* actuation, because the property verified is physical and unobservable until the command is attempted; the safe state is, therefore, reached by reversal rather than refusal, the only construction available when the evidence of failure is the absence of an expected response.

Analogous but shorter gating applies at B6 (a feed is accepted only if provenance-verified and within TTL, otherwise it requires local corroboration or human approval before changing the plan) and at B3 (an override is authorised only if the session is MFA-verified and originates from a recognised context, otherwise it is blocked regardless of token validity). The negative control implements neither algorithm: telemetry is used as received, commands actuated unconditionally without feedback check, feeds applied directly, overrides honoured on token presentation, and updates installed regardless of signature or rollout policy.

#### 4.2.1. Experimental Setup

The six attack and failure scenarios plus the nominal check were reproduced as automated injections and run once against each controller under identical initial conditions, each in isolation. Every run records the evidence fields: authentication result, freshness delta, sequence and replay result, measured feedback flow, MFA and session state, signature and rollout status. This constitutes exactly what a real deployment’s logging subsystem would need for audit and investigation.

Table 15 summarises the outcome of each scenario against both controllers. An outcome is unsafe if the injection achieved its objective, such as an unauthorised or physically unverified actuation, an unvetted schedule change, or an unverified firmware install, and safe/blocked if the architecture prevented it or bounded the system to a defined safe state.

As the secure controller is designed to implement exactly the checks required to defeat the six scenarios, the separation below shows that the mechanisms are *internally consistent* with the scenarios they were designed against and with the claims of Section 2.4 and Section 2.6. Because the first six scenarios are attack cases, they establish only *sensitivity*; the seventh submits five legitimate operations with no injected fault, establishing *specificity*.

Figure 10 presents the same outcomes visually, separating the six attack scenarios, which establish sensitivity, from the nominal-operation control, which establishes specificity.

#### 4.2.2. Linking Runtime Evidence to the Provenance Model

**Figure A1:**
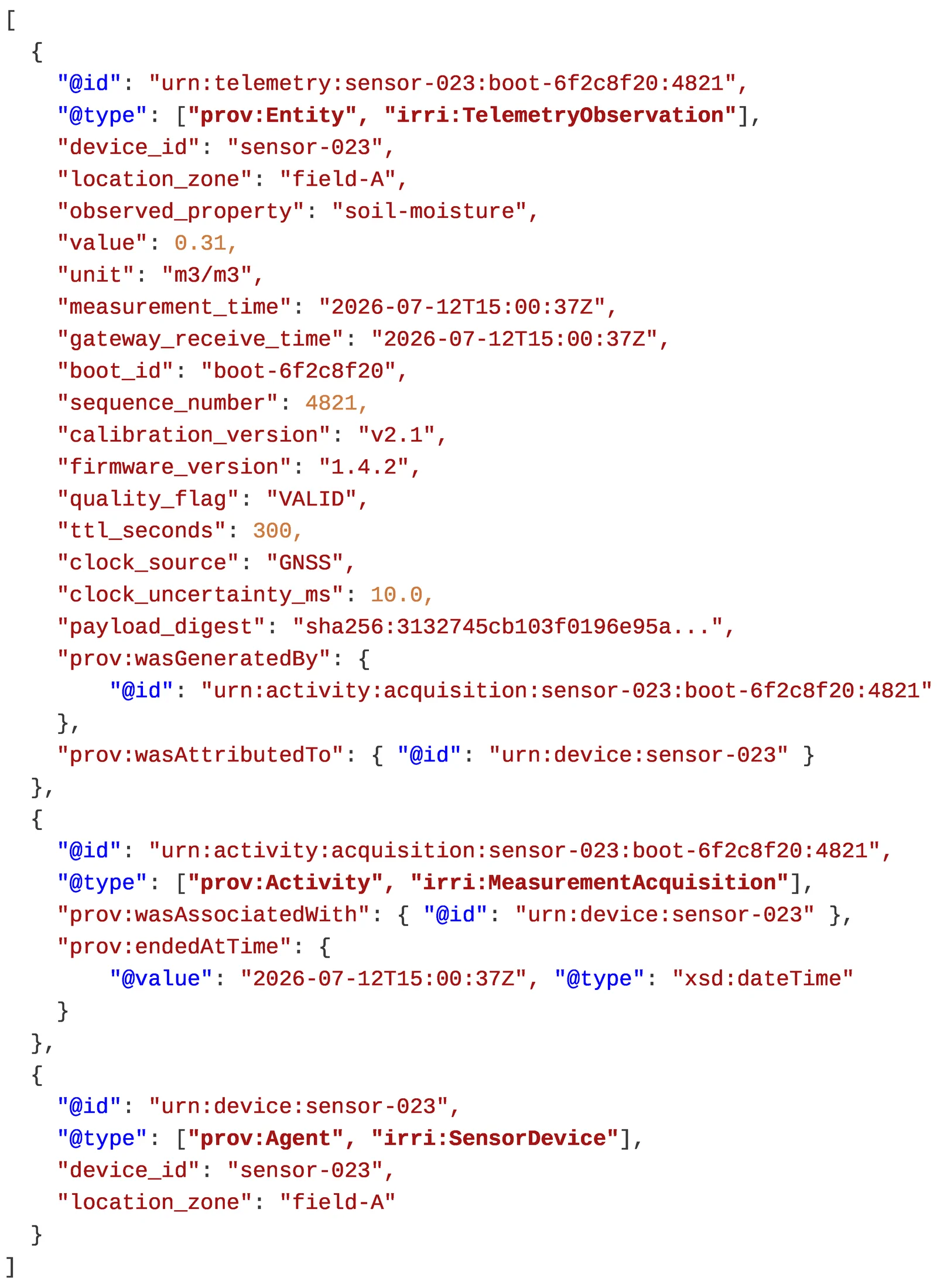
PROV-O/JSON-LD provenance record minted by the secure controller for an ACCEPTED telemetry observation.

The provenance requirements of Section 2.6.1 were exercised directly rather than merely specified. For every record passing Accept(r), the secure controller mints a PROV-O/JSON-LD record following the profile. Critically, a record that *fails*Accept(r), such as the forged reading in the spoofed-telemetry scenario, never has a provenance entity minted at all: its absence from the log is itself auditable evidence rather than a separately logged error that could be edited out. Appendix A, Figure A1, reproduces a record from the actual run, precisely from sensor-023, and immediately before the spoofed reading was injected, carrying all elements named in Section 2.6.1: record identifier, device identity and zone, observed property/value/unit, measurement and gateway-receive timestamps, boot identifier and sequence number, calibration and firmware versions, quality flag, TTL, clock source and uncertainty, and payload digest, linked to its acquisition activity and originating agent through prov:wasGeneratedBy and prov:wasAttributedTo.

The forged record that follows in the same scenario fails the authentication check in Algorithm A1 and is returned as Invalid and produces *no* corresponding prov:Entity, demonstrating in one run both the positive case (accepted observation → auditable provenance chain) and the negative case (forged observation → marked Invalid, no provenance, no effect on the irrigation decision) required by the verification criteria.

#### 4.2.3. Result Verification

Because the results of Table 15 are generated entirely by code, they are falsifiable rather than asserted. Three mechanisms support this. Execution is deterministic and self-contained: no network calls, no random number generator affecting any pass/fail decision, and no dependency beyond the Python standard library, so the script reproduces identical outcomes on any machine with Python ≥ 3.9. Output is machine-readable: the script writes poc_results.json with every scenario’s controller, outcome and evidence trail, plus the run’s Coordinated Universal Time (UTC) timestamp, Python version, platform string, and the Secure Hash Algorithm 256 (SHA-256) hash of the script itself (SHA-256: 78f2ba975c6c9cee783741f98d66b8ae5c16dca934b866f109d48a7ef4ef40d3). And the script asserts its outcomes rather than printing them: naive_unsafe_count = 6, secure_unsafe_count = 0 and secure_false_positive_count = 0 are checked in code, and the process exits non-zero if any assertion fails, so a run terminating with exit code 0 is itself evidence that the claimed separation was reproduced. Together these establish the reproducibility of the reported run.

### 4.3. Quantitative Overhead of the Architecture

The scenario results establish that the controls behave as specified within the model; they say nothing about cost. Because irrigation deployments are constrained by radio duty cycle, battery and gateway storage more often than by processing power, that question is frequently decisive. The figures are derived analytically from the record structure of Section 2.6.1 and the LoRa time-on-air model [91,92], applied to the LoRaWAN frame format [93]: exact consequences of the stated encoding, reproducible from the parameters given.

#### 4.3.1. Per-Message Overhead

A minimal unauthenticated application payload carrying a measurement and timestamp occupies 6 bytes: a 2-byte scaled/fixed-point sensor reading plus a 4-byte compact (Unix-epoch) timestamp. The fields Accept(r) require adding, in compact binary encoding, a device identifier (4 B), boot identifier (2 B), sequence number (4 B), quality flag (1 B), TTL (2 B) and a truncated hash-based message authentication code tag computed with SHA-256 (HMAC-SHA-256, 8 B): a 27-byte authenticated application payload, an increase of 21 bytes. On air, each payload is carried in a LoRaWAN frame that adds a fixed 13 bytes of medium access control (MAC) framing (a 1-byte MAC header (MHDR), 7-byte frame header (FHDR), 1-byte frame port (FPort) and 4-byte message integrity code (MIC) [93]), so the transmitted frames are 19 bytes unauthenticated and 40 bytes authenticated. The 4-byte device identifier is an application-layer field bound cryptographically under the HMAC tag. It is deliberately re-asserted rather than inferred from the LoRaWAN device address (DevAddr): the DevAddr is covered by the network-layer MIC [93], but it is a network-assigned, non-permanent session address verified with a network-server key, and, therefore, not a stable, application-bound device identity on which the gateway’s Accept(r) check can rely. Verbose serialisation is far more costly (66 bytes as compact JavaScript Object Notation (JSON), 631 bytes as the full JSON-LD provenance entity, roughly 9.6 times the compact form), which is the quantitative justification for a rule now made explicit: *the provenance graph is minted at the gateway, not transmitted from the field device*. Deployments that transmit JSON-LD from an endpoint over a low-power link will exhaust their duty-cycle allowance before their battery.

#### 4.3.2. Airtime and Duty Cycle

Table 16 gives the consequence for LoRaWAN at 125 kHz bandwidth, coding rate (CR) 4/5, explicit header and 8-symbol preamble, computed with the time-on-air formula of [91] on the full 19-byte and 40-byte LoRaWAN frames, with sustainable uplink rates under the 1% duty cycle of the European Union (EU) 868 MHz band  [94,95]; comparable capacity limits are analysed in [96]. Relative cost falls as the spreading factor (SF) rises (60% at SF7 to 50% at SF12) because the preamble, header and fixed MAC framing amortise the added payload, but absolute cost rises sharply: at SF12 the security fields cost 655 ms per message and the sustainable interval lengthens from 132 s to 197 s. Full per-record authentication is, therefore, affordable at SF7–SF9 for sampling intervals of minutes, covering most soil-moisture and flow telemetry, whereas a deployment at SF12 must lengthen its interval, batch observations under one authentication tag, or accept lower device density. The architecture accommodates all three, because the requirement is that freshness and integrity be re-established at B1, not per reading; batching shifts the granularity, which must then be reflected in the TTL bound.

#### 4.3.3. Gateway Storage

At 631 bytes per record and a 15-min interval, one sensor generates 96 records per day (about 59 KiB); 100 devices accumulate roughly 5.8 MiB per day and 2.1 GiB per year before compression. This is negligible for a gateway with a modest solid-state disk and material for one running from internal flash, which is the binding case. Where it binds, two-tier retention is permitted: full entities held locally long enough for incident investigation, then reduced to identifier, digest and acceptance outcome (about 96 bytes, or 321 MiB per year) with full entities archived off-gateway. The audit property that matters, that a rejected record has no provenance entity and that this absence is evidence, survives the reduction.

#### 4.3.4. Latency

The latency added by the enforcement points is dominated not by cryptography but by the feedback window, which is bounded below by the hydraulic transport delay of the zone and is, therefore, a property of the plumbing rather than the software; for loops operating over seconds to minutes it is immaterial. The exception is fast-acting protective functions such as pressure relief or emergency shutoff, where feedback verification must not sit in the trip path: the architecture requires the protective action to be executed unconditionally and verified afterwards, inverting the ordinary order.

### 4.4. Applying the Architecture: A Hypothetical Worked Example

A reference architecture is useful only if the path from model to installation is clear. This section works through it for a *hypothetical* drip-irrigated orchard in six hydraulic zones, with 24 soil-moisture probes, 6 zone valves, 2 pressure transducers, a main-line flow meter, a pump with a variable-frequency drive, one field gateway, a cloud scheduler with a weather subscription, and a mobile dashboard used by two operators and a contractor, small enough to be realistic and complex enough to exercise all six boundary classes. All parameter values below are illustrative assumptions.

*Step 1: enumerate assets and assign trust levels.* Each component is mapped to a class from Table 8 and scored with the rubric. Probes are A1 in the T1–T2 band; the low-cost units are additionally Tier H2, which fixes their verification regime. Valves, pump and drive are A2 at T3 by E1; the gateway A3 at T3 by E3 then E1; the cloud scheduler A4b at T3 and the historian A4a at T2; the dashboard A5 in T2–T3 and the device credentials and firmware signing key A6 at T4 by E2, inventoried separately from the gateway using them. The output is a spreadsheet of about forty rows whose value is to make visible how few assets, about a dozen of the forty, hold T3 or T4 authority and so warrant the strongest controls.

*Step 2: locate the boundary crossings.* Probe-to-gateway telemetry crosses B1; gateway-to-cloud buffered telemetry crosses B2; dashboard and mobile overrides cross B3; the LoRaWAN field segment, the cellular backhaul, and the operator’s public-internet path are three distinct B4 crossings; the valve cabinets and the gateway enclosure are B5 surfaces and the weather subscription and contractor remote-support access are B6. Table 2 then specifies what must be re-established per crossing, and Table 6 specifies which requirement each control discharges.

*Step 3: set the deployment-specific parameters.* A 15-min sampling interval with k=2 and a 20-s worst-case transmission delay gives a 35-min telemetry TTL; the command TTL is 30 s, short enough that a captured command cannot usefully be replayed after an operator leaves the field; the feedback window is 45 s, assuming a representative fill time for the longest lateral; minimum expected flow for an open valve is 60% of nominal, tolerating partial clogging without masking a stuck-closed valve; offline autonomy is bounded at 24 h, after which the gateway completes any running cycle and switches to the conservative fallback programme of Section 2.6.4 under independent interlocks until an operator confirms; a zone held closed after a feedback fault reverts to that programme after a deficit-tolerance window of 12 h in mid-season, shortened during fruit sizing; the gateway takes time from NTS-authenticated NTP cross-checked against GNSS, and command freshness falls back to gateway-issued nonces once the tracked clock uncertainty exceeds 2 s; provenance retention is 90 days local, digest-only thereafter.

*Step 4: choose the maturity level.* The deployment adopts the minimum baseline in full, plus the two elements disproportionately valuable relative to cost: feedback-aware execution, needing only that the existing flow meter be read within the window, and per-record authentication at SF7–SF9, affordable at the chosen interval. Full PKI with automated certificate lifecycle is deferred in favour of pre-shared keys with documented rotation, an explicitly recorded risk acceptance.

*Step 5: verify against the acceptance criteria.* In a conforming physical deployment, the seven scenarios of Table 5 *would be* executed against the installed system before handover, using the injection methods listed there. The pass criterion is not that the system resists the attack in some general sense but that the specific evidence named in the final column is present in the logs, which converts commissioning from an inspection into a test.

The sequence is deliberately mechanical, and that is its purpose: the architecture is meant to be executable by an integrator rather than interpreted by a researcher. Residual judgement is concentrated in step 3, and the sensitivity analysis of Section 2.5 bounds how much the judgement in step 1 can matter.

### 4.5. Generalisation to Other Cyber-Physical Agriculture Settings

Irrigation serves as the case study because it exhibits the full difficulty compactly: constrained outdoor sensing, an actuator with immediate physical consequence, a slow and noisy feedback channel, and a legitimate remote-control requirement. The invariant structure, consisting of an exposed sensing layer, a local enforcement point with authority over actuation, crossings at which trust assumptions change, and a physical feedback signal against which commands can be verified, is not specific to it, and any agricultural cyber-physical system with that structure maps onto the model.

Table 11 sets out the substitution for four adjacent settings. The boundary and crown-asset classes are unchanged throughout; what changes is the feedback channel, the process time constant, and the consequence of an unsafe action. Two settings are more demanding than irrigation. Fertigation and chemical dosing invert the safety asymmetry: for irrigation the conservative failure mode is usually to stop, whereas an interrupted dosing cycle can leave an unbalanced application, so the bounded safe state must be defined per operation rather than globally. Livestock watering makes availability itself the safety property, since a stopped supply is harmful within hours, so failing closed, which is appropriate for a valve that might flood a field, is exactly wrong. The architecture accommodates both through the definition of the bounded safe state; what the model contributes is the requirement that the parameter be defined and justified rather than inherited by default, following the graded procedure of Section 2.6.4.

The one setting in which the architecture does not transfer unmodified is autonomous field machinery, where the actuator is mobile, the safety envelope is defined by proximity to people rather than a hydraulic limit, and the enforcement point must reason about a changing environment. The trust-boundary analysis carries over, but feedback-aware execution would need extending from verifying that a commanded action occurred to verifying that it occurred within a dynamically computed safe envelope. This is a boundary of applicability rather than future work.

## 5. Discussion

This section interprets the results of Section 4. In summary they are four: the mechanisms are internally coherent with the architecture’s own acceptance criteria, separating 6/6 from 0/6 unsafe outcomes with no false positive on legitimate operation; their cost is bounded at 21 bytes and 50–60% additional time-on-air per record, conditional on where the provenance graph is built; the classification is stable under 78% of single-step disagreements, fully stable for the actuator class and stable under 6 of 7 perturbations (86%) for the gateway/RTU/PLC class and the model can be applied step by step to a hypothetical deployment and transfers to four adjacent settings, with the physical feedback channel, process time constant, and bounded safe state as the setting-specific parameters.

### 5.1. Verification Results

The results show a clear separation rather than a partial improvement, and every scenario compromising the negative control does so through the *absence* of one specific control rather than a sophisticated exploit: an unauthenticated value, an expired timestamp, an unconfirmed physical action, an unverified session context, a stale feed, an unsigned artefact. Within the scope stated in Section 2.1, the separation supports the central argument: irrigation security is not primarily a cryptography or network-protection problem but a question of whether every crossing re-establishes identity, freshness, authorisation and evidence appropriate to the receiving function.

Three outcomes merit attention. The feedback-timeout scenario shows authorisation checks alone are *not sufficient* for actuator safety: the command is fully authorised, and the unsafe outcome is detected and reversed only by the independent feedback-verification step, confirming feedback-aware execution as a requirement distinct from authorisation. The stale-feed and compromised-session scenarios show that boundary-specific controls generalise the same Accept()-style reasoning beyond telemetry to schedule inputs and human actions, supporting the claim that all six classes need structurally similar, risk-proportionate enforcement rather than ad hoc rules. And the nominal scenario shows the discrimination is not achieved by indiscriminate rejection: presented with five legitimate operations, the secure controller accepted all of them, exactly as the negative control did, addressing the methodological risk that a controller rejecting everything would trivially also score 0/6.

The quantitative analyses carry their own implications. Cost is dominated by a single design choice, where the provenance graph is constructed, rather than by cryptography: a 21-byte authenticated uplink is affordable across the spreading factors used for field telemetry, whereas transmitting the 631-byte entity from the endpoint is not. The controls are, therefore, compatible with constrained deployments, but only under a specific division of labour between endpoint and gateway, which the analysis makes explicit rather than leaving implementers to discover. The sensitivity analysis is complementary: the actuator class (A2), which has direct authority over the physical process, is fully stable; the gateway/RTU/PLC class (A3), which also has direct authority, is stable under 6 of 7 perturbations (86%) and changes only when its credential/update authority reaches the trust-anchor threshold itself, the condition under which it should be re-inventoried at T4, while classes whose authority is indirect are markedly less stable, and for these the escalation rules rather than the score should govern. The model’s subjective element is thus concentrated where its consequences are smallest.

### 5.2. Comparative Analysis of the Integrated Framework

Table 3 compared the architecture with existing approaches on declared capabilities, necessarily before results existed. Now that the framework has been executed, quantified and applied, a more demanding comparison is possible: not which capabilities each approach claims, but what each delivers as an integrated whole and what evidence it offers. Table 17 does this for six frameworks a practitioner might realistically adopt instead, along dimensions assessable only after the results: the object actually protected, whether conformance can be decided, whether the cost of conformance is stated, whether the framework’s own judgements are shown robust, whether an executable artefact accompanies it, and whether transfer beyond the original setting is mapped explicitly.

Three observations follow, of which the third is a concession. First, the framework is distinguished less by any individual capability than by the closure of the loop between them. Each comparator is stronger on at least one axis: specifically, IEC 62443 has an assessment ecosystem this model lacks; fault-tree analysis yields failure probabilities it does not attempt; ledger-based provenance gives cross-organisational non-repudiation a gateway-minted record cannot. What none does is connect a stated requirement to the boundary at which it must be re-established, to the asset whose authority makes it matter, to a criterion that decides conformance, and to an analytically calculated cost. The value of the integration is that a claim in Section 2.2 traces forward to a pass/fail decision in Table 15 and a byte count in Table 16; a framework supplying one link leaves the others to the adopter.

Second, the comparison isolates one capability that none of the frameworks in Table 17 delivers: treating a command as incomplete until the physical process confirms it. These frameworks terminate their reasoning at the authorisation decision. The underlying idea is not new: safety instrumented systems, process-aware intrusion detection, control invariants and physics-based anomaly detection all use physical consistency as a signal (Section 1.1.3). Those mechanisms, however, act as parallel detection or protection layers rather than as a completion condition inside the command path. In a hydraulic process, the difference matters, and the feedback-timeout scenario is the demonstration: the command passes every authorisation check, and the unsafe state is reached anyway. The contribution in this respect is, therefore, the integration of physical confirmation into the per-command enforcement point and its evidence trail, not the concept of physical verification itself.

Third, the framework is weaker than several comparators on the dimension mattering most for adoption, external verification. IEC 62443 conformance can be assessed by a party with no stake in the outcome; these acceptance criteria have so far been evaluated only by their authors against an implementation written by their authors. Table 17 should, therefore, be read as a comparison of what each framework specifies and evidences, not a claim of demonstrated superiority in the field. Converting the criteria into an independently administered conformance procedure, possible in principle, since each one names the evidence a log must contain, is the step that would make the comparison fair.

### 5.3. Implications for Secure Digital Farming

The architecture reframes secure digital farming as a trustworthy cyber-physical operation problem rather than a purely technical cybersecurity task, and its main implication is that security cannot be assigned to a single layer or mechanism. Encrypted telemetry is insufficient if the data are stale, unauthenticated or lack provenance; a secure dashboard is insufficient if it can trigger irrigation without local interlocks; cloud authentication is insufficient if a gateway queue can reintroduce expired commands; a signed firmware update assures security only alongside rollback protection, traceability and audit evidence. The architecture also shows why availability and resilience are first-class security objectives here: connectivity loss, packet loss, battery depletion, sensor drift, actuator malfunction and hydraulic faults are ordinary operating conditions, so the system must degrade predictably through bounded autonomy, local fallback rules, safe defaults, store-and-forward logging, manual override and defined recovery workflows rather than failing into ambiguous behaviour and because a safe default that protects equipment can harm the crop, and one that protects the crop can waste water or stress the hydraulics, the default itself must be chosen and time-bounded per zone (Section 2.6.4).

A further implication concerns cost and scalability. Large enterprises can deploy PKI, segmentation, SIEM integration, redundant communications and formal incident response; small farms often need simpler first steps such as asset inventory, unique credentials, restricted remote access, watering limits, manual override and basic alarms. By separating the logical requirement from the implementation mechanism, the architecture supports both.

### 5.4. Limitations

Four limitations bound these results. First, and stated once here for the whole paper: the results evidence the architecture’s logical soundness rather than a deployment’s performance. Every result of the paper is one of three kinds: a synthesis of existing standards and guidance into a taxonomy (Section 2.2, Section 2.3 and Section 2.4); a simulation whose separation of outcomes follows from how the secure controller was specified to enforce exactly the checks under test, compared against an intentionally unsecured negative control rather than an adaptive attacker or an independently engineered baseline (Section 4.2); or an analytical calculation from the published LoRa time-on-air model applied to the LoRaWAN frame format [91,93] (Section 4.3). The simulation abstracts away protocol timing, cryptographic hardware constraints and network conditions, and none of the three kinds is a new empirical measurement. Under the design-science framing of Section 2.1, what the combination contributes is an integration no single cited source performs (Table 1 and Table 3), a formal specification shown to be internally consistent and executable, which is a necessary precondition for field evaluation rather than a substitute for it, and a cost estimate reproducible from stated parameters rather than measured on hardware. Whether that combination constitutes a sufficient contribution in the absence of field data is a legitimate point on which reviewers may reasonably disagree; the position taken here is that it is a defensible, explicitly bounded first step, and the testbed of Section 5.5 is what would clear the empirical bar. Second, deployment-specific parameters are left open by design (specifically the command TTL, offline autonomy, pressure thresholds, response windows, log retention, safe-state definitions), which follows from the technology-neutral scope but means adopting the model does not by itself guarantee a safe deployment; in particular, the deficit-tolerance windows of the graded safe state (Section 2.6.4) depend on crop, growth stage, soil and climate, and only stage (i) of that procedure is exercised by the proof-of-concept. Third, the trust rubric and the STRIDE ratings were applied by the authors rather than by independent raters, and no inter-rater agreement figure is reported; the anchors are stated explicitly enough to be re-applied, and the escalation rules bound how much one rater’s judgement can affect a directly-actuating or credential-issuing asset, but this remains structured expert judgement rather than a validated instrument. Likewise, the requirements R1–R9 derive from a manual, author-selected literature search for which no screening log was kept; a different selection of sources might yield a different requirement set.

Fourth, a field deployment would expose transients that the plausibility model of Section 2.7 cannot fully exercise. Examples include genuine moisture drops caused by preferential/macropore flow, rapid increases from wetting fronts after irrigation or rainfall, apparent diurnal moisture cycles caused by the temperature dependence of the dielectric and electrical properties on which capacitive and resistive probes rely, and probe–soil interface changes from shrink-swell cycling in clay-rich soils. Section 2.7 routes these cases through corroboration witnesses C1–C3 and the SUSPECT state of Section 2.6.2, but the corresponding rate limits and agreement bands are stated rather than field-calibrated. As noted in Section 5.5, calibration of these thresholds by soil type and season would transform this architectural provision into a tested one. Likewise, the time-source, drift, reboot, counter-loss and recovery provisions of Section 2.6.3 are specified but not injected in the proof-of-concept.

### 5.5. Future Research

Future work should implement the architecture in a physical or simulated testbed, ideally evaluated independently of the design team, measuring latency, energy, communication overhead, false-positive rates, fault tolerance and operational safety under the same scenarios, and thereby testing against an adaptive attacker and third-party baselines rather than only the intentionally unsecured negative control, and establishing values for the parameters left open. A separable direction concerns the classifications of Section 2.5 and Section 3.1: external analysts should independently apply the rubric of Table 7, and analogously the STRIDE anchors, to a shared asset set, reporting inter-rater agreement and a reconciliation protocol, which would convert the rubric into a validated instrument. A third direction follows from Section 5.2: because each acceptance criterion names the evidence a deployment’s logs must contain, the criteria could become an independently administered conformance procedure, in the manner of an IEC 62443 assessment, closing the one gap on which this framework is weaker than the standards-based approaches it is compared against. A fourth, more immediate direction is field calibration of the plausibility bounds in Section 2.7: logging soil-moisture, temperature and hydraulic traces through irrigation and rainfall events at a real site would show how often preferential/macropore flow, a passing wetting front, the temperature dependence of probe readings and shrink-swell air-gap formation trigger the SUSPECT state of Section 2.6.2 and would let the C1–C3 thresholds be tuned per soil type and season instead of left at the single generic rate limit used here. A fifth direction is to extend the scenario set with time and state faults: clock drift over long isolation, GNSS or NTP spoofing, gateway counter loss, epoch replay with a reused boot identifier, and recovery after prolonged disconnection, each with acceptance criteria derived from Section 2.6.3. A sixth is to derive deficit-tolerance windows for the graded safe state of Section 2.6.4 from crop water-stress data and to evaluate, in a coupled hydraulic and crop model, the yield and water cost of fail-closed, fail-open and graded policies under attack and fault.

## 6. Conclusions

This paper set out to determine what a reference architecture for smart irrigation must specify if the security of the system is to be assessable rather than asserted, and whether the resulting specification is coherent, affordable, and applicable.

Within the design-stage scope stated in Section 2.1, three results follow. First, the architecture is internally coherent against its own acceptance criteria: the intentionally unsecured negative control was compromised in all six attack scenarios, the secure controller in none, and the separation was not produced by indiscriminate rejection, since the secure controller executed all five legitimate operations of the nominal control with no false positives. The feedback-timeout scenario is decisive: a fully authorised command still produced an unsafe outcome, which only independent physical confirmation detected and reversed, making feedback-aware execution a requirement distinct from command authorisation rather than a refinement of it. Second, the architecture is affordable within the constraints that actually bind agricultural deployments: per-record authentication costs 21 bytes and 50–60% additional time-on-air depending on spreading factor, sustainable at typical sampling intervals provided the provenance graph is minted at the gateway, and provenance retention costs about 2.1 GiB per year for 100 devices, reducible by a factor of about six (to approximately 321 MiB per year) under digest-only retention without losing the audit property that a rejected record leaves no provenance entity. Third, the architecture is applicable rather than only descriptive: 78% of single-step perturbations leave the classification unchanged, with the actuator class fully insensitive and the gateway/RTU/PLC class insensitive under 6 of 7 perturbations (86%), changing only when it acquires trust-anchor authority, the path from model to installation is a five-step procedure illustrated through a hypothetical worked deployment example, and the layer, boundary and asset structure transfers unchanged to greenhouse climate control, fertigation, livestock watering and cold-chain storage, with the physical feedback channel, process time constant, and bounded safe state as the setting-specific parameters.

The architecture rests on two principles the results support: local safety authority, keeping final actuation permission at the field gateway, RTU or PLC-like controller, and feedback-aware execution, treating a command as complete only when the physical process confirms it. Their combined effect is that a digital compromise anywhere above the local enforcement point cannot produce an unverified physical action, provided that the enforcement point’s time and counter state are protected (Section 2.6.3) and that its bounded safe state is chosen per zone to balance equipment, water and crop (Section 2.6.4). The principal limitation, stated in Section 5.4, is that this evidence is architectural rather than empirical. Closing that gap through an independently evaluated testbed, and converting the trust rubric into a validated instrument through an inter-rater study, are the immediate next steps.

## Figures and Tables

**Table 1:** Summary of the literature analysis: established results, residual gaps, and the corresponding response in this paper.

Reviewed Strand	What Is Established	Residual Gap for Secure Irrigation	Response in This Paper
CPS in smart agriculture (Section 1.1.1)	Layered, data-driven irrigation and greenhouse architectures are feasible and improve water use; closed-loop sensing and actuation are well characterised.	Architectures enumerate components but do not state where trust assumptions change, nor what must be re-established at a crossing.	Seven-layer decomposition with three planes and six boundary classes (Section 2.3 and Section 2.4).
Edge control and OT (Section 1.1.2)	Edge controllers reduce cloud dependency and sustain operation during wide area network (WAN) loss; legacy OT protocols lack native authentication.	Local autonomy is treated as an availability feature rather than a security enforcement point with authority over actuation.	The local safety authority principle (Section 2.6), enforced in Algorithm A2.
Threat modelling (Section 1.1.3)	STRIDE-style and fault-tree analysis identify attack surfaces and failure propagation in agricultural IoT.	Threats are not connected to irrigation crown assets, nor to the boundary at which the control must act.	STRIDE asset×boundary matrix (Table 4) over the A1–A6 inventory.
Provenance, authentication, lifecycle (Section 1.1.4)	Ledger- and public key infrastructure (PKI)-based mechanisms give tamper-evident records and secure update paths.	Provenance serves downstream traceability rather than admission control on the telemetry driving a decision.	Machine-checkable Accept(r) and PROV Ontology (PROV-O) profile, with provenance minted only for accepted records.
Verification practice (all strands)	Proposals are typically argued analytically or shown on a single protocol.	Few architectures state what evidence would falsify them, so conformance cannot be assessed at procurement.	Scenario-based acceptance criteria (Table 5) and a deterministic proof-of-concept.

**Table 2:** Trust-boundary classes, transitions, risks, and required enforcement patterns for the proposed secure smart-irrigation architecture.

Boundary	Transition	Change in Trust Assumptions	Main Risks	Required Controls
**B1: Field–Edge Boundary**	From field sensors and actuator states to the gateway, RTU, or PLC.	Data originates from exposed, constrained, and physically accessible field devices.	Spoofed or replayed soil-moisture values, sensor tampering, calibration loss, packet loss, and battery depletion.	Device authentication, anti-replay counters or nonces, freshness windows, plausibility checks, calibration and location metadata, and tamper-aware logging.
**B2: Edge–Platform Boundary**	From local OT devices to brokers, registries, ingestion APIs, or cloud services.	Local OT state and buffered events move into platform or cloud environments.	Queue poisoning, stale telemetry, reintroduced old commands, and loss of queue integrity.	Mutual authentication, scoped service identity, queue integrity, store-and-forward semantics, rate limiting, and immutable ingest logs.
**B3: Platform–Application Boundary**	Between analytics services, rule engines, dashboards, HMIs, and mobile applications.	Decisions, views, approvals, and overrides move between platform services and human or application operators.	Compromised dashboard tokens, unsafe overrides, stale views, and confused-deputy behaviour.	MFA, RBAC/ABAC, session protection, separation of duties, reason codes, bounded overrides, and full operator audit.
**B4: Connectivity-Zone Boundary**	Across low-power wide-area network (LPWAN), Wi-Fi, cellular, Ethernet, OT/IT, Internet, VPN, or DMZ segments.	Flows cross zones with different exposure, latency, reliability, and administrative control.	Man-in-the-middle attacks, replay, denial of service, uncontrolled remote access, and lateral movement.	Zones and conduits, firewall policy, private APN/VPN where appropriate, protocol allowlisting, monitoring, redundancy, and controlled remote access.
**B5: Physical-Tamper Boundary**	Physical access to sensors, valves, cabinets, debug ports, or controllers.	Trust is affected by the possibility of physical manipulation or extraction of secrets.	Sensor manipulation, bypassed controllers, credential extraction, and debug/Joint Test Action Group (JTAG) interface abuse.	Tamper-evident enclosures, locked debug ports, secure boot, protected secrets, hardware identity where feasible, and incident logging.
**B6: Third-Party / External-Feed Boundary**	From weather feeds, satellite inputs, vendor APIs, contractors, or remote support interfaces to the irrigation system.	External sources with different trust levels may influence operational decisions.	Stale weather feeds, over-privileged vendor access, unreliable third-party data, and unsafe schedule changes.	Provenance checks, TTL/freshness limits, least privilege, scoped access, explicit approval for high-impact decisions, and local corroboration.

**Table 3:** Comparison of the proposed reference architecture with representative existing approaches to securing smart-irrigation and agricultural cyber-physical systems. • = addressed explicitly; ∘ = partially or implicitly addressed; − = not addressed.

Approach	Primary Lens	Layered Architecture	Explicit Trust Boundaries	Asset-Level Trust Classes	Local Safety Authority	Feedback-Aware Execution	Testable Accept. Criteria
IoT-enabled smart-agriculture architectures [10,12,13]	Functional/system design	•	−	−	−	−	−
IEC 62443 mapping for agricultural IoT [4]	Device security levels, zones and conduits	∘	∘	∘	−	−	∘
Threat modelling/attack-surface analysis of agricultural IoT [39]	Adversary and attack surface	−	∘	−	−	−	−
Model-based safety analysis with fault trees [40]	Component failure propagation	−	−	∘	∘	∘	∘
Safety instrumented systems, process-aware and physics-based ICS monitoring [41,42,43,44,45,46,47]	Physical consistency of commands and process	−	−	−	∘	∘	∘
Zero-trust architectures for industrial and manufacturing settings [58,59]	Access control posture	∘	•	−	−	−	−
Authentication and tamper-resistant schemes for agricultural IoT [37,38]	Cryptographic protocol	−	∘	−	−	−	∘
Blockchain-based provenance and traceability [48,49,50]	Data integrity and ledger	−	∘	−	−	−	−
Edge-computing irrigation controllers [31]	Predictive control at the edge	∘	−	−	∘	−	−
This paper	Trust-boundary crossings in a cyber-physical process	•	•	•	•	•	•

**Table 4:** STRIDE-based threat matrix across irrigation crown assets (rows) and trust boundaries (columns). Each cell gives the dominant STRIDE categories and the qualitative impact rating. STRIDE: S = Spoofing, T = Tampering, R = Repudiation, I = Information disclosure, D = Denial of service, E = Elevation of privilege. Impact: L = Low, M = Medium, MH = Medium–High, H = High, C = Critical.

Asset Class	B1 Field–Edge	B2 Edge–Platform	B3 Platform–App.	B4 Connectivity	B5 Physical-Tamper	B6 Third-Party
A1 Telemetry sources	S, T, D H	T, R, I, D MH	I, R M	S, T, I, D MH	S, T, D H	S, T, R MH
A2 Actuators	T, D H	S, T, R H	S, T, R, E H	T, D H	T, D, E H	T, E MH
A3 Local/edge controllers	S, T, D, E H	S, T, R, D, E **C**	S, T, R, E H	T, D, E H	T, I, E **C**	S, T, R MH
A4 Cloud/platform services	S, T, D MH	S, T, R, I, D, E H	R, I, E H	I, D, E H	I, D M	S, T, R H
A5 Dashboards, HMI, mobile	R, I L–M	T, R, I M	S, T, R, E H	I, D, E MH	S, I, E MH	I, E H
A6 Identities and credentials	S, E H	S, T, E **C**	S, R, E **C**	I, D, E H	I, E **C**	S, E **C**

**Table 5:** Scenario-based verification tests for the proposed architecture.

Scenario	Injection Method	Expected Architectural Response	Evidence Required for Pass/Fail Decision
Spoofed telemetry	False soil-moisture value from an unauthorised identity, or replay of an old valid packet.	Gateway rejects the value or marks it low-trust; it is not used for autonomous actuation.	Authentication result, counter/nonce mismatch, freshness status, reason code, unchanged decision.
Command replay	Reissue an old valve-open command after its TTL or sequence number has expired.	Local controller refuses execution and records a duplicate or expired command.	Command identifier, TTL result, controller decision, actuation status, operator alert.
Feedback timeout	Command valve opening while blocking expected flow or pressure feedback.	Controller detects command-feedback asymmetry and triggers alarm or safe shutdown.	Command timestamp, feedback window, sensor readings, interlock action, final safe state.
Compromised operator session	High-impact override using a valid but stolen dashboard token.	Additional MFA, context-bound or dual approval required; unsafe action blocked.	Session metadata, requester role, approval status, blocked command record, audit trail.
Stale external feed	Weather data older than the configured freshness limit.	Feed marked stale; local corroboration or human approval required before the plan changes.	Feed timestamp, source identity, TTL evaluation, decision trace, corroboration status.
Unsafe update	Unsigned firmware, or signed firmware outside approved rollout policy.	Device rejects the update or rolls back; failure logged and alerts raised.	Signature verification result, version metadata, rollback event, affected assets, operator notification.
Nominal operation (specificity check)	Submit well-formed, legitimate telemetry, a command, an operator override, a weather feed, and a firmware update, with no injected fault (all correctly authenticated, fresh, in-policy, and MFA-verified where applicable).	Gateway/controller accepts and actuates/executes all five operations normally; none is incorrectly rejected.	Authentication/freshness/ordering results, feedback-verification result, MFA/session state, feed provenance/freshness result, update signature/rollout result, each confirming acceptance, not rejection.

**Table 6:** Mapping from reviewed sources to derived requirements, implementing layers, and the trust-boundary classes at which each requirement must be re-established.

ID	Requirement	Principal Sources	Implementing Layer(s)	Boundaries
R1	Unique, verifiable device and workload identity	OT asset inventory and IoT identity baselines [1,61,63]	Field sensors; gateway/RTU/PLC; edge-cloud platform; management and governance	B1, B2, B6
R2	Message integrity and origin authentication	OT guidance; agricultural authentication schemes [1,37,38]	Field sensors; gateway/RTU/PLC; connectivity zones	B1, B2, B4
R3	Freshness and replay resistance	Industrial control system (ICS) engineering controls; documented replay attacks on smart farms [4,7]	Gateway/RTU/PLC; connectivity zones	B1, B2, B4, B6
R4	Authorisation proportionate to physical authority	Zero-trust and separation-of-duties practice [14,58,59]	Applications and operations; gateway/RTU/PLC	B3, B6
R5	Local enforcement under degraded connectivity	Edge-control and OT autonomy literature [1,31,33]	Gateway/RTU/PLC	B1, B2, B4
R6	Independent physical confirmation of actuation	Closed-loop irrigation control and safety analysis [2,5,20,40]	Actuators and physical process; gateway/RTU/PLC	B1, B5
R7	Machine-readable provenance and non-repudiable evidence	World Wide Web Consortium (W3C) PROV-O practice; agricultural traceability work [48,50,53]	Edge-cloud platform; gateway/RTU/PLC	B1, B2, B6
R8	Secure lifecycle governance	Secure-development and update guidance [57,67,68,69]	Management and governance; edge-cloud platform	B2, B5, B6
R9	Bounded, predictable degradation under failure	OT safe-state practice; fault-tree analysis of irrigation [1,40]	Actuators; gateway/RTU/PLC; connectivity zones	B1, B4, B5

**Table 7:** Trust-level scoring rubric: criteria, 0–3 anchors, and threshold mapping to T0–T4.

Scoring Criterion (0–3 Each)	Anchor Description
Physical actuation authority	0 = none; 1 = indirect influence via a recommendation the local controller may reject; 2 = can request actuation subject to local gating; 3 = can directly command a valve, pump, relay, or VFD.
Decision influence	0 = none; 1 = one input among several corroborated sources; 2 = a primary input to a scheduling/policy decision; 3 = can unilaterally trigger or suppress an irrigation action.
Credential or update authority	0 = none; 1 = holds a scoped, short-lived credential; 2 = holds a long-lived service credential or can push configuration to a bounded asset set; 3 = can sign code/firmware, issue or revoke credentials, or modify trust anchors.
Exposure to compromise	0 = not reachable outside a managed, physically secured network; 1 = reachable only via an internal segmented network; 2 = reachable via a public or shared network path; 3 = physically accessible in an unmonitored outdoor/remote location.
Consequence of failure	0 = negligible operational effect; 1 = degraded visibility or reporting; 2 = degraded reliability or unsafe irrigation under compounding conditions; 3 = unsafe irrigation, equipment damage, or loss of control integrity on its own.
**Total Score**	**Default Trust Level (Before Escalation)**
0–2, and asset is external/unmanaged	T0
0–4	T1
5–8	T2
9–12	T3
13–15	T4

**Table 8:** Irrigation-centric crown assets, trust levels, and minimum controls.

ID	Asset Type	Irrigation Function and Safety Impact	Trust Level	Minimum Controls	Typical Failure or Misuse Modes
A1	Telemetry sources: soil, flow, pressure, weather, valve-state sensors	Generate measurements that trigger, suppress or validate decisions; incorrect or stale values cause wrong watering or missed failures.	T1–T2	Source authentication, integrity, freshness, location and calibration metadata, secure onboarding, plausibility checks.	Replay, spoofing, drift, calibration loss, packet loss, battery depletion, DoS, physical tamper.
A2	Actuators: valves, relays, pumps, VFDs, fertigation units	Execute physical actions; incorrect commands cause over-irrigation, dry running, overpressure or dosing errors.	T3	Strict authorisation, command integrity, TTL/freshness, interlocks, safe defaults, signed configuration in the controlling path.	Stuck-open/stuck-closed state, replayed open command, bypassed interlock, unsafe manual override.
A3	Local/edge controllers: RTU, PLC, microcontroller unit (MCU), gateway	Mediate measurements, policies and physical action; execute local logic; operate during WAN loss.	T3	Mutual authentication, policy enforcement, secure boot, attestation where feasible, signed updates, local historian, rollback.	Logic tampering, queue poisoning, stale state, unsafe normalisation, compromised gateway.
A4	Cloud/platform services: broker, registry, ingestion API, historian, analytics, scheduler	Coordinate data collection, decision support, rules, orchestration and software distribution.	T2–T3	Service identity, scoped authorisation, immutable ingest logs, provenance anchoring, rate limiting, signed artefacts.	Misconfiguration, stale models, over-privileged services, compromised registry, unsafe scheduler.
A5	Dashboards, HMI, mobile operations	Provide visibility, approval, manual override, alarms, and incident response.	T2–T3	MFA, RBAC/ABAC, session protection, reason codes, bounded override, immutable operator audit.	Token theft, stale views, confused deputy, unsafe override, lack of dual approval.
A6	Identities and credentials: PKI, pre-shared keys (PSK), tokens, signing keys	Establish who may communicate, approve, update, recover, or prove operational events.	T4	Lifecycle governance, rotation, revocation, hardened storage, break-glass policy, audit of issuance and use.	Key leakage, PSK reuse, token replay, signer compromise, undocumented recovery access.

**Table 9:** One-at-a-time sensitivity analysis of the trust-level classification. Criterion scores are (physical actuation authority, decision influence, credential/update authority, exposure, consequence of failure). “Stable” counts the admissible single-anchor-step perturbations that leave the final trust level unchanged.

Asset	Scores	Total	Default Band	Rule Applied	Final	Stable Perturbations
A1 Telemetry sensor	(0, 2, 0, 3, 2)	7	T2	decision tree	T1–T2	7/7 (100%)
A2 Actuator (valve, pump)	(3, 1, 0, 3, 3)	10	T3	E1	T3	6/6 (100%)
A3 Gateway/RTU/PLC	(3, 3, 2, 2, 3)	13	T4	E3 then E1	T3	6/7 (86%)
A4a Analytics/historian	(0, 1, 1, 2, 1)	5	T2	—	T2	5/9 (56%)
A4b Scheduler/registry	(2, 3, 2, 2, 2)	11	T3	—	T3	8/9 (89%)
A5 Dashboard/HMI	(2, 1, 1, 2, 2)	8	T2	—	T2–T3	5/10 (50%)
A6 PKI, signing keys	(0, 0, 3, 1, 3)	7	T2	E2	T4	5/6 (83%)
**Total**						**42/54 (78%)**

**Table 10:** Functional layers of the proposed secure digital-farming architecture.

Layer	Role in Smart Irrigation	Minimum Security and Resilience Requirements
Field sensors and telemetry	Measure soil moisture, flow, pressure, weather, water level, valve state.	Device identity, source authentication, integrity and freshness checks, calibration metadata, plausibility checks, tamper evidence, safe degradation under packet loss.
Actuators and physical process	Execute physical actions via valves, relays, pumps, VFDs, fertigation units.	Strict command authorisation, local interlocks, bounded runtime/pressure/volume limits, safe defaults, manual override, command-feedback verification.
Field gateway/RTU/PLC	Mediate telemetry and actuation; run local policies; normalise and buffer data; operate during WAN loss.	Mutual authentication, policy enforcement, secure boot, signed configuration, local historian, queue integrity, rollback, backup power, local safety authority.
Connectivity and network zones	Transport telemetry, commands, logs and updates across field, farm, OT, IT and cloud segments.	Zones and conduits, virtual local area network (VLAN)/demilitarised zone (DMZ)/virtual private network (VPN) or private access point name (APN), quality of service (QoS), redundancy, anti-replay, controlled remote access, monitoring.
Edge/cloud platform	Ingestion, registries, brokers, analytics, scheduling, decision support, update distribution.	Workload identity, scoped authorisation, secrets management, rate limiting, signed artefacts, immutable ingest logs, provenance anchoring, resilience to cloud failure.
Applications and operations	Dashboards, HMI, mobile access, approvals, reporting, alarms, manual overrides.	Multi-factor authentication (MFA), role-based/attribute-based access control (RBAC/ABAC), session protection, separation of duties, reason codes, dual approval for high-impact actions, operator audit trail.
Management and governance	Identities, PKI, asset inventory, firmware lifecycle, vulnerability handling, backups, incident response.	Certificate lifecycle, key rotation and revocation, software bill of materials (SBOM) and supplier requirements, secure update governance, incident runbooks, retention rules, recovery testing.

**Table 11:** Mapping of the reference architecture to adjacent cyber-physical agriculture settings. The layer, boundary, and crown-asset structure is invariant; the physical feedback channel, process time constant, and definition of the bounded safe state are setting-specific.

Setting	Sensed Quantities (A1)	Actuators (A2)	Physical Feedback for Verification	Process Time Constant	Bounded Safe State
Drip and sprinkler irrigation (this paper)	Soil moisture, flow, pressure, valve state	Zone valves, pump, VFD	Flow rise, pressure change, valve transition, soil-moisture response	Seconds to hours	Graded per zone (Section 2.6.4): immediate hold (close valves, stop pump), then a conservative pre-approved fallback programme under independent interlocks
Greenhouse climate control	Air temperature, humidity, CO_2_, radiation, vent position	Vents, heaters, foggers, screens, CO_2_ injection	Rate of change of the controlled variable within the expected envelope	Minutes	Vent to ambient, disable injection, maintain frost protection
Fertigation and chemical dosing	Electrical conductivity, pH, tank level, injector state	Dosing pumps, injectors, mixing valves	Conductivity and pH response downstream of the injection point	Seconds to minutes	Complete or safely terminate the dosing cycle; never leave a partial application
Livestock watering and cooling	Trough level, flow, water temperature, ambient heat index	Supply valves, pumps, cooling sprinklers	Level recovery after draw; flow at the point of use	Minutes to hours	Fail *open* to a bounded supply; availability is the safety property
Post-harvest storage and cold chain	Temperature, humidity, door state, ethylene	Refrigeration, humidifiers, ventilation	Temperature trajectory against the thermal model of the loaded store	Hours	Maintain last safe setpoint; alarm rather than switch off

**Table 12:** Trade-offs between fail-closed, fail-open, and graded safe-state policies in irrigation.

Policy	Protects	Harms or Creates	Architectural Mitigation
Fail-closed (close valves, stop pump, hold)	Pump against dry running; pipework against overpressure and water hammer; soil against flooding and erosion; water budget; fertigation dose limits	Crop water stress and irreversible yield loss if prolonged in heat or at sensitive growth stages; salt accumulation where leaching is missed; exploitable as a denial-of-irrigation attack	Hold per zone rather than per farm; bound the hold by a crop- and season-specific deficit-tolerance window; alarm immediately
Fail-open (continue last schedule or leave supply open)	Crop or livestock water supply; continuity through sensor or link faults	Water waste and allocation breaches; waterlogging, nitrate leaching and runoff; unobserved bursts or stuck-open valves; pump overpressure or dry running if supply or downstream state changes	Permit only under interlocks independent of the failed channel (pressure switch, pump-current or run-dry trip, maximum runtime and metered volume) and for a bounded duration
Graded, time-bounded (this paper)	Equipment first; crop after the tolerance window	Residual deficit stress during stage (i); residual water use during stage (ii)	Stage (i) hold, stage (ii) conservative pre-approved programme under independent interlocks, stage (iii) operator confirmation logged as a B3 event; protective trips unconditional throughout

**Table 13:** Physical sensor fault modes, their observable signatures, and the edge-control and trust-boundary responses they dictate. The final column indicates whether the fault is distinguishable from a deliberate attack using the corroboration witnesses of Section 2.6.2.

Sensor Class	Dominant Physical Fault Modes	Observable Signature	Required Edge Verification (B1)	Attack/Fault Discrimination
Capacitive/resistive soil moisture	Electrode corrosion, salinity and fertigation interference, air gap at the probe–soil interface	Slow monotonic offset; step change on disturbance; abnormally low variance if detached	Calibrated range per soil type, physical rate limit, per-epoch SUSPECT rate, mandatory spatial corroboration for Tier H2	Drift fails C2 progressively but passes C1; spoofing fails C3 immediately
Pressure transducer	Thermal zero-drift, diaphragm fatigue, water-hammer overrange, blocked impulse line	Baseline offset correlated with temperature; flat-lined reading; transient spikes	Temperature-compensated band, cross-check against pump state and flow, rate-of-change limit	Strong: hydraulic consistency (C3) is directly observable
Flow meter (mechanical or ultrasonic)	Impeller fouling, air entrainment, signal attenuation, partial-pipe conditions	Under-reading at nominal pressure; intermittent dropout	Mass-balance check against valve state and pressure; minimum expected flow gate	Strong: flow is the feedback channel, so failure shows as command–feedback asymmetry
Valve position/limit switch	Contact wear, mechanical binding, stuck-open or stuck-closed actuator	State reported without corresponding flow change	Command–feedback verification within the feedback window; interlock on disagreement	Strong: disagreement is the detection criterion regardless of cause
Weather and environmental (temperature, humidity, rain)	Radiation-shield ageing, sensor wetting, thermal lag, siting error	Diurnal bias; implausible saturation; slow response to step changes	TTL-bounded use, corroboration against the external feed at B6, advisory-only classification	Moderate: local and external sources corroborate one another
Low-cost aggregate (Tier H2)	All of the above, with wider unit-to-unit variation and no internal reference	Higher baseline SUSPECT rate; larger inter-device spread within a zone	Tightened plausibility band from datasheet accuracy, gateway-assigned time and sequence, default spatial corroboration, never a sole actuation input	Weakest individually; recovered by redundancy rather than device quality

**Table 14:** Derivation of the verification scenarios: the boundary crossed, the assets involved, the single architectural control under test, and the dominant STRIDE categories from Table 4.

Scenario	Boundary	Assets	Sole Control Under Test	STRIDE	Claim Falsified If It Fails
S1 Spoofed telemetry	B1 field–edge	A1, A3	Source authentication and integrity within Accept(r)	S, T	That B1 re-establishes origin before a value can influence actuation
S2 Command replay	B2 edge–platform, B4 connectivity	A2, A3	Command TTL and replay/sequence check in local safety authority	S, T, D	That freshness is re-established at every crossing, not only at ingest
S3 Feedback timeout	B1 field–edge, physical process	A2, A1	Independent physical feedback verification	T, D	That feedback-aware execution is a requirement distinct from authorisation
S4 Compromised operator session	B3 platform–application	A5, A6, A2	MFA and context binding for high-impact overrides	S, R, E	That a valid session is insufficient authority for a physical action
S5 Stale external feed	B6 third-party	A4, A1	Provenance and TTL corroboration of external inputs	S, T, R	That external data cannot change the irrigation plan uncorroborated
S6 Unsafe firmware update	B2 edge–platform, B5 physical-tamper	A3, A6	Signature verification and rollout-policy enforcement	T, E	That lifecycle governance is enforced in the control path, not administratively
S7 Nominal operation	none (control)	A1–A6	Specificity check	–	That the controls discriminate rather than reject indiscriminately

**Table 15:** Proof-of-concept verification results: intentionally unsecured negative control vs. secure controller (CTRL) under the six Table 5 attack scenarios plus a seventh, nominal-operation specificity check. Flow is given in litres per minute (lpm).

Scenario	Negative Control	CTRL(Secure)	Key Evidence (Secure Controller)
Spoofed telemetry (B1)	Unsafe: forged low soil-moisture value accepted, irrigation triggered	Blocked	authenticated=False (but integrity_valid=True: the forged payload is well-formed, just unsigned by a device key); record rejected, irrigation decision unchanged
Command replay (B2/B4)	Unsafe: expired valve-open command executed	Blocked	ttl_ok=False (age 600 s > ttl 30 s); command rejected before actuation
Feedback timeout (B1)	Unsafe: valve marked OPEN with no physical confirmation	Blocked (safe state)	flow=0.0 lpm< 5.0 lpm expected; command-feedback asymmetry → alarm, valve forced CLOSED
Compromised operator session (B3)	Unsafe: override executed on stolen token alone	Blocked	mfa_verified=False, issuer_ip_known=False; override blocked pending MFA
Stale external weather feed (B6)	Unsafe: 3-h-old forecast applied directly to schedule	Blocked	fresh=False (age 10,800 s > ttl 1800 s); requires local corroboration
Unsafe firmware update (B2/B5)	Unsafe: unsigned image installed without checks	Blocked	signed=False, signature_valid=False; update rejected, alert raised
Nominal operation (no injected fault)	Negative control: all five legitimate operations executed as submitted (no check performed)	Accepted/Executed	All five legitimate operations accepted: authenticated, fresh, ordered, quality_ok all True (record accepted, provenance minted); signature_valid, ttl_ok, replay_ok True with feedback flow 42.0≥5.0 lpm (command executed and feedback-verified); mfa_verified, issuer_ip_known True (override authorised); provenance_ok, fresh True (feed accepted); signed, signature_valid, approved_rollout True (update installed)
**Sensitivity (6 attack scenarios)/Specificity (1 nominal scenario)**			
neg. control unsafe: **6/6 (100%)**	secure unsafe: **0/6 (0%)**	lneg. control false pos.: **0/1 (0%)**	secure false pos.: **0/1 (0%)**

**Table 16:** Analytically derived time-on-air and duty-cycle overhead of per-record authentication over LoRaWAN, computed with the formula of [91] on full frames, including the 13-byte LoRaWAN MAC overhead [93] (bandwidth (BW) 125 kHz, CR 4/5, explicit header, 8-symbol preamble, cyclic redundancy check (CRC) on, low-data-rate optimisation at SF12). Sustainable uplink rates assume the 1% duty-cycle limit of the EU 868 MHz band [94,95].

Spreading Factor	Time-on-Air, 19 B Frame (6 B Payload)	Time-on-Air, 40 B Frame (27 B Payload)	Relative Overhead	Sustainable Uplinks per Hour (Baseline → Authenticated)
SF7	51.5 ms	82.2 ms	+60%	699 → 438
SF9	185.3 ms	287.7 ms	+55%	194 → 125
SF12	1318.9 ms	1974.3 ms	+50%	27 → 18

**Table 17:** Comparative analysis of the integrated framework against comprehensive security reference architectures and methodologies for cyber-physical agriculture and adjacent domains, along dimensions assessable only after the results of Section 4. • = delivered with supporting evidence; ∘ = partially delivered or asserted without evidence; − = not addressed.

Framework	Object Protected	Decidable Conformance	Cost of controls Quantified	Robustness of Own Judgements Shown	Executable Artefact	Transfer Mapped	Distinctive Strength Retained over This Paper
IEC 62443 applied to agricultural IoT [4]	Zones and the devices within them	•	−	−	−	∘	Certification ecosystem and independent third-party assessment
OT security guidance, NIST SP 800-82r3 [1]	The control system as an organisational asset	∘	−	−	−	•	Breadth across governance, workforce, and physical security
Zero-trust architectures for industrial settings [58,59]	Access to resources	∘	∘	−	∘	•	Mature identity and policy-engine tooling
Threat modelling and attack-surface analysis [39]	The attack surface	−	−	−	−	∘	Adversary-centric coverage independent of any one architecture
Model-based safety analysis with fault trees [40]	Component failure propagation	∘	−	∘	∘	∘	Quantitative failure probabilities, which this paper does not provide
Blockchain provenance and traceability frameworks [32,48,49,50]	The record, after the fact	∘	∘	−	•	•	Cross-organisational non-repudiation without a trusted party
**This paper**	**The physical process, through the crossings that govern it**	•	•	•	•	•	**—**

**Figure A2:**
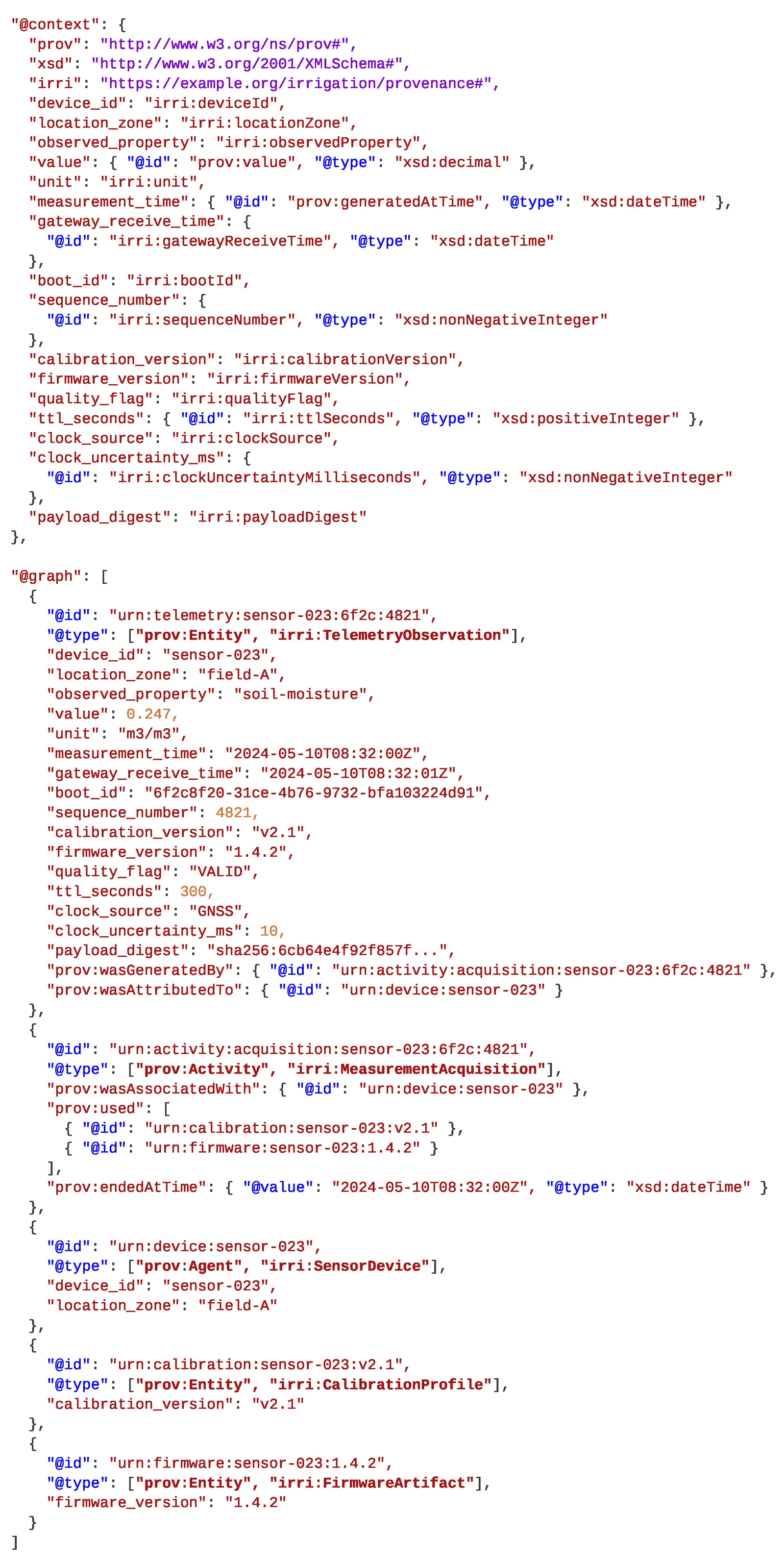
Structure of the JSON-LD/PROV-O context and telemetry-observation graph used to encode the minimum provenance record defined in Section 2.6.1.

## Data Availability

The proof-of-concept source code, the data and the results produced for this work are available through the GitHub repository [97].

## Abbreviations

The following abbreviations are used in this manuscript:

ABAC	Attribute-Based Access Control
API	Application Programming Interface
APN	Access Point Name
BW	Bandwidth
CI/CD	Continuous Integration and Continuous Delivery
CoAP	Constrained Application Protocol
CPS	Cyber-Physical System
CPU	Central Processing Unit
CR	Coding Rate
CRC	Cyclic Redundancy Check
DevAddr	Device Address (LoRaWAN)
DMZ	Demilitarised Zone
ETSI	European Telecommunications Standards Institute
EU	European Union
FHDR	Frame Header (LoRaWAN)
FPort	Frame Port (LoRaWAN)
GNSS	Global Navigation Satellite System
HMAC	Hash-based Message Authentication Code
HMI	Human–Machine Interface
HTTPS	Hypertext Transfer Protocol Secure
ICS	Industrial Control System
IEC	International Electrotechnical Commission
IoT	Internet of Things
IR	Interagency/Internal Report (NIST)
ISO	International Organisation for Standardisation
JSON	JavaScript Object Notation
JSON-LD	JavaScript Object Notation for Linked Data
JTAG	Joint Test Action Group (debug/test interface)
LoRa	Long Range (radio modulation)
LoRaWAN	Long Range Wide Area Network
lpm	Litres per minute
LPWAN	Low-Power Wide-Area Network
MAC	Medium Access Control
MCU	Microcontroller Unit
MFA	Multi-Factor Authentication
MHDR	MAC Header (LoRaWAN)
MIC	Message Integrity Code
MQTT	Message Queuing Telemetry Transport
NB-IoT	Narrowband IoT
NIST	National Institute of Standards and Technology
NTS	Network Time Security
NTP	Network Time Protocol
OPC UA	Open Platform Communications Unified Architecture
OT	Operational Technology
PKI	Public Key Infrastructure
PLC	Programmable Logic Controller
PoC	Proof of Concept
ppm	Parts per million
PRISMA	Preferred Reporting Items for Systematic Reviews and Meta-Analyses
PROV-O	PROV Ontology
PSK	Pre-Shared Key
PTP	Precision Time Protocol
QoS	Quality of Service
RBAC	Role-Based Access Control
REST	Representational State Transfer
RTC	Real-Time Clock
RTU	Remote Terminal Unit
SBOM	Software Bill of Materials
SF	Spreading Factor
SHA-256	Secure Hash Algorithm 256
SHACL	Shapes Constraint Language
SIEM	Security Information and Event Management
SP	Special Publication (NIST)
STRIDE	Spoofing, Tampering, Repudiation, Information Disclosure, Denial of Service, Elevation of Privilege
TTL	Time to Live
UTC	Coordinated Universal Time
VFD	Variable-Frequency Drive
VLAN	Virtual Local Area Network
VPN	Virtual Private Network
W3C	World Wide Web Consortium
WAN	Wide Area Network

## Appendix A. Serialised Provenance Records

This appendix reproduces the JSON-LD serialisations referenced in Section 2.6.1 and Section 4.2.2. They are placed here, rather than in the body, because they are normative reference material for implementers: the argument of the paper depends on the *structure* of the provenance record, which is stated formally in Section 2.6.1, and not on any particular serialisation of it. Figure A1 shows a record minted by the secure controller during the run reported in Section 4.2, for a telemetry observation that passed Accept(r). The forged record injected immediately afterwards in the same scenario produces no counterpart to Figure A1: its absence from the provenance log is the auditable evidence of its rejection. Figure A2 shows the JSON-LD context together with the telemetry-observation graph that encodes the minimum provenance record of Section 2.6.1.

## Appendix B. Pseudocode for the Enforcement Points

This appendix gives the pseudocode corresponding to the two decision flows presented in Figure 11 and Figure 12. Algorithm A1 is the data-plane enforcement point at boundary B1, and Algorithm A2 is the control-plane enforcement point that implements local safety authority with feedback verification. The PoC of Section 4.2 implements both directly.

**Algorithm A1** Telemetry acceptance, Accept(r)
Require: telemetry record $r=(\mathrm{device}\_\mathrm{id},\mathrm{value},t_{m},t_{g},\mathrm{boot}\_\mathrm{id},\mathrm{seq},\mathrm{ttl},\mathrm{sig},\mathrm{quality}\_\mathrm{flag})$ Ensure: Accepted, Suspect, or Invalid (Accept(r)=true iff the return value is Accepted; Suspect and Invalid are both instances of ¬Accept(r), distinguished here for downstream corroboration and logging) 1: auth←VerifyHMAC(sig,deviceKey[device_id],Canonical(*r*)) 2: $\mathrm{fresh}\leftarrow \left(\left(t_{g}-t_{m}\right)\in \left[-u_{r},\;\mathrm{ttl}+u_{r}\right]\right)$ 3: lastSeq←LastSeq(device_id,boot_id) 4: ordered←(seq>lastSeq) 5: **if** ¬(auth∧fresh∧ordered) **then** 6: **return** Invalid ▹artefact-level failure; no provenance entity minted; MUST NOT affect the irrigation decision 7: **end if** 8: LastSeq(device_id,boot_id)←seq ▹state advances only once auth/fresh/ordered hold 9: **if** quality_flag=Valid **then** 10: **return** Accepted ▹record may drive irrigation decision 11: **else** 12: **return** Suspect ▹ provenance retained; enters corroboration of Figure 7; MUST NOT independently trigger or suppress a high-impact irrigation action 13: **end if**

**Algorithm A2** Local safety authority with feedback verification, ExecutedCommand(c)
Require: command $c=(\mathrm{command}\_\mathrm{id},\mathrm{action},t_{\mathrm{issued}},\mathrm{ttl},\mathrm{seq},\mathrm{issuer},\mathrm{sig})$ Ensure: Executed or Rejected/SafeState 1: sigOk← Verify HMAC(sig,issuerKey[issuer], Canonical (c)) 2: $\mathrm{ttlOk}\leftarrow \left((\mathrm{now}-t_{\mathrm{issued}})\leq \mathrm{ttl}\right)$ 3: replayOk←(command_id∉seenIds)∧(seq>lastCmdSeq) 4: **if** ¬(sigOk∧ttlOk∧replayOk) **then** 5: **return** Rejected ▹ rejected before any actuation occurs 6: **end if** 7: seenIds←seenIds∪{command_id}; lastCmdSeq←seq 8: Actuate(action) ▹ e.g., open valve 9: **if** action= OpenValve **then** 10: $\mathrm{feedbackOk}\leftarrow \left(\mathrm{measuredFlow}\geq \mathrm{MIN}\_\mathrm{EXPECTED}\_\mathrm{FLOW}\right)$ within FEEDBACK_WINDOW 11: **if** ¬feedbackOk **then** 12: Actuate(Stopvalve) ▹force bounded safe state 13: RaiseAlarm(command_feedback_asymmetry) 14: **return** SafeState 15: **end if** 16: **end if** 17: **return** Executed
